# Dropout Rate of Participants in Randomized Controlled Trials Using Different Exercise-Based Interventions in Patients with Migraine. A Systematic Review with Meta-Analysis

**DOI:** 10.3390/healthcare13091061

**Published:** 2025-05-05

**Authors:** Sahar Taghipourazam, Maria-Dolores Cortes-Vega, Cristina García-Muñoz

**Affiliations:** 1 Department of Physiotherapy, University of Seville, 41009 Seville, Spain; saharttpp@gmail.com; 2 Departamento de Ciencias de la Salud y Biomédicas, Universidad Loyola Andalucia, 41704 Seville, Spain; cgmunoz@uloyola.es; 3 CTS 1110: Understanding Movement and Self in Health From Science (UMSS) Research Group, 41009 Andalusia, Spain

**Keywords:** migraine, exercise, dropout rate, adherence, systematic review, meta-analysis

## Abstract

**Background/Objectives:** Exercise has gained attention as a potentially beneficial non-pharmacological intervention, but whether this type of intervention presents a higher dropout rate compared to other interventions is still unknown. This systematic review, with a meta-analysis of randomized controlled trials, aims to determine whether exercise or comparators present lower or higher attrition in patients with migraine. **Methods:** A search was conducted in PubMed, Scopus, Web of Science, and Cochrane Library until March 2025. The methodological quality was evaluated using the JBI scale for randomized trials. Proportion meta-analysis calculated the dropout rate. **Results:** Odds ratio meta-analysis under 1 indicated lower attrition in experimental participants. Subgroup meta-analyses sorted by type of exercise, control, and migraine were conducted to explore variability in results based on the mentioned moderators. The overall pooled dropout rate was 6.7%, 11.6% for the exercise groups, and 10.1% for the comparators. No statistical difference was found between groups of studies, type of migraine, type of exercise, and type of comparator (*p* ≥ 0.05). Only the odds ratio results for migraine with auras showed a lower pooled dropout rate in favor of control participants, OR = 1.18. **Conclusions:** Although there is no statistically significant difference, the meta-analysis of proportions shows a higher loss rate in exercise-based interventions. However, the high heterogeneity found in the included studies prevents us from drawing firm conclusions. Furthermore, adequate adherence to the CONSORT guidelines in reporting losses and their reasons could help design appropriate retention strategies for studies and interventions based on exercise in patients with migraines.

## 1. Introduction

Globally, migraine is recognized as the sixth most significant and disabling condition that people experience throughout their lives, becoming a barrier in various aspects of daily life [1]. According to the World Health Organization (WHO), migraine is a primary headache disorder, typically episodic, lasting between 4 and 72 h. It is often accompanied by nausea, vomiting, and/or sensitivity to light (photophobia) and sound (phonophobia). In some cases, it is preceded by a short-duration aura consisting of unilateral, reversible visual, sensory, or other symptoms [2].

Nowadays, there are plenty of pharmacological options available for migraineurs, but at the same time, patients’ lack of adherence to the use of prophylactic medicines cannot be denied [3]. The limited availability of effective treatment options for managing migraines highlights the fact that there is still much we do not understand about the underlying causes and mechanisms of this condition. However, recent research highlights the effectiveness of non-pharmacological approaches in managing migraine attacks and controlling pain [4].

Exercise is one of the popular approaches for controlling chronic pain situations and common pathologies like sleeping disorders or psychological problems [4]. Considering the benefits of this intervention, it can be chosen as a non-pharmacological treatment for migraines. Nevertheless, contrary to all the points mentioned, there are some migraine patients who report an increase in their headaches after starting regular physical activity following exercise protocols [3]. This has led to physical inactivity, inability to engage in physical movement, and prolonged sedentary behavior becoming common symptoms among individuals affected by this pathology [5]. Migraine is a complex neurological disorder involving both peripheral and central sensitization mechanisms. Peripherally, the activation of the trigeminovascular system leads to the release of vasoactive neuropeptides and neurogenic inflammation. Centrally, brainstem dysfunction alters descending pain modulation. Exercise may influence these mechanisms by inhibiting nociceptive input at the spinal dorsal horn and enhancing central pain inhibitory pathways [6,7]. Moreover, physical activity patterns vary across migraine phases, with marked hypoactivity during attacks, suggesting that structured interventions should be phase-adapted [8]. Given the heterogeneity of migraine, with variability in attack frequency, aura, treatment response, and comorbidities, a multimodal and individualized approach is increasingly recommended. Recent studies support the role of slow aerobic exercise and physiotherapy in reducing symptom burden and enhancing neural mechanisms involved in pain modulation, such as insular and cingulate connectivity [9,10]. All these findings strengthen the rationale for integrating tailored exercise into migraine care.

Despite the potential benefits of exercise in reducing migraine frequency and intensity, adherence to exercise programs among individuals with migraines remains highly controversial. While some studies support the hypothesis that exercise can serve as a protective factor and a preventive approach to migraine attacks, others indicate that it may act as a trigger, exacerbating symptoms in certain individuals [3]. This dual effect contributes to the inconsistency in adherence levels, as patients may either benefit from exercise as a long-term management strategy or avoid it out of fear of worsening their symptoms [11].

Despite growing evidence supporting the benefits of exercise in migraine management, there is a lack of clarity regarding adherence to such interventions, particularly concerning dropout rates and their underlying causes. Understanding these patterns is critical, as dropout can limit the effectiveness and real-world applicability of exercise-based treatments. This systematic review aims to answer the following research question: What is the overall dropout rate in randomized controlled trials involving exercise interventions for migraine, and how does it compare with control interventions? Based on the previous literature, we hypothesize that exercise-based interventions may present slightly higher dropout rates, potentially due to the paradoxical effects of physical activity acting as both a therapeutic tool and a symptom trigger in certain individuals. Considering the previous information and the importance of different exercise protocols in controlling migraine pain and also the lack of systematic reviews with the aim of analyzing the reasons behind the dropouts that happen among the participants in exercise protocols to control migraine pain, we propose a systematic review of randomized clinical trials that use various exercise protocols as a non-medical intervention for this pathology, with the aim to meta-analyze the dropout rate in each and the reasons behind each one of them to know what the burdens on the way of migraineurs patients to use this type of intervention for treating their condition are.

## 2. Materials and Methods

### 2.1. Data Sources and Search Strategy

This systematic review was developed in accordance with the 2020 Preferred Reporting Items for Systematic Reviews and Meta-Analyses (PRISMA 2020) statements [12]. The review protocol was registered in the OSF registry with DOI 10.17605/OSF.IO/NUHTQ. Two independent reviewers (ST and MDCV) conducted an independent systematic search in the different databases. A systematic search was conducted in the following databases: PubMed, Scopus, Web of Science, and Cochrane Library to identify studies analyzing the impact of different types of exercise on migraine or control in patients with migraines from the inception of databases to March 2025. No predefined filters were applied during the initial search, but specific keywords such as migraine, chronic migraine, episodic migraine, aerobics, and yoga, among others, were used to get more accurate results. Search terms were combined using Boolean operators such as AND or OR. The search strategy is shown in detail in Supplemental Material S1.

### 2.2. Research Question and Study Selection

The PICOS (Population, Intervention, Comparators, Outcomes, and Study Design) model was used to build the eligibility criteria for this systematic review [13].

The inclusion criteria were as follows:

P: Adult participant between 18 and 65 years old with any type of migraine;

I: Exercise interventions (e.g., aerobics, yoga, tai chi, relaxation, endurance, and strength, among others);

C: Any type of comparator, except those based on exercise;

O: Participants’ dropout;

S: Randomized clinical trials that report the dropout rate or number of participants who withdrew or studies that allow its indirect calculation.

The exclusion criteria were as follows:

- Conference papers;
- Dissertations.

### 2.3. Data Extraction and Quality Assessment

After the independent search, the same reviewers (ST and MDCV) carried out the screening and selection by title and abstract. Potential articles were managed using Mendeley desktop version 1.19.8, and duplicates were removed using this citation manager. In cases of disagreements, a third reviewer (CGM) was consulted. When a potential manuscript could not be accessed, or there were missing data, the corresponding authors were contacted. A list of excluded studies and their reasons is available in Supplementary Material S2.

Data of interest were recorded in a table of characteristics and an EXCEL spreadsheet for the meta-analysis. The extracted data were author, year, sample, participant characteristics (age, sex, type of migraine), experimental/control intervention description, overall retention rate, dropout rates by group, reasons for dropout, and adverse events.

The methodological quality of studies was assessed using the Joanne Brigs Institute tool (JBI) for randomized clinical trials [14]. This tool was designed for use in systematic reviews; it is also useful for critical appraisal of individual trials in clinical and research settings. Its application contributes to a better understanding of the available evidence and more informed decision-making in evidence-based practice. The JBI for randomized clinical trials is composed of 13 items that are scored as “yes”, “no”, “uncertain”, or “not applicable”. This scale is exempt from an overall score. The results were compiled in a table format.

In cases where data on dropout rates or reasons for withdrawal were not available in the included articles, the authors were contacted directly to gather the necessary information via e-mail. After the selection of studies, no corresponding author was contacted.

Certainty of evidence was assessed using the GRADE system. This system allows for the evaluation of five domains: (i) risk of bias, (ii) inconsistency, (iii) indirectness, (iv) imprecision, and (v) level of evidence. Randomized controlled trials begin with a “high” level of evidence, but domains could be downgraded.

### 2.4. Data Analysis

A participant was considered a dropout if he/she did not complete the study intervention or follow-up period following the randomization process. Comparisons between groups were conducted separately in a pairwise manner for studies that included more than two intervention groups.

For the quantitative analysis, R Studio software (version 4.1.2) was utilized, employing the metafor, meta, and dmetar packages [15,16,17]. All meta-analyses were performed using a random-effects model, assuming heterogeneity among the included randomized clinical trials. The corresponding forest plots illustrated the meta-analysis results. A proportion meta-analysis was conducted to estimate both the overall pooled dropout rate and the pooled dropout rate for each intervention group. Proportions were transformed using the logit transformation [18]. As some studies did not report dropout cases, a continuity correction of 0.5 was applied [19]. Additionally, a meta-analysis based on the odds ratio (OR) was performed to examine dropout patterns across intervention groups. An OR < 1 indicated a lower attrition rate among participants who received exercise training. To assess the effect measure, the 95% confidence interval (95% CI) was calculated, and data were adjusted using the inverse variance method for sparse data [20]. The restricted maximum likelihood estimator for Tau^2^ was employed to estimate between-study variance. Study heterogeneity was assessed using the I² statistic, with values exceeding 50% considered significantly high [21]. The OR meta-analysis was conducted to estimate both the overall pooled dropout rate and the dropout rate pattern for each intervention group.

Outliers were identified through sensitivity analysis, which included exploratory techniques generating Baujat, L’abbé, leave-one-out, and Influence plots. Studies detected as influential in sensitivity analysis and potentially introducing bias were excluded from the meta-analysis. Furthermore, publication bias was assessed using a contour-enhanced funnel plot, incorporating the trim-and-fill method for adjustment, alongside Egger’s tests for bias detection (presence of publication bias when *p* < 0.05) [22].

To identify how the type of migraine, type of exercise, and control comparator affected the overall pooled effects, subgroup meta-analyses displayed in forest plots were conducted.

## 3. Results

### 3.1. Study Selection and Methodological Quality Assessment

**Figure 1:**
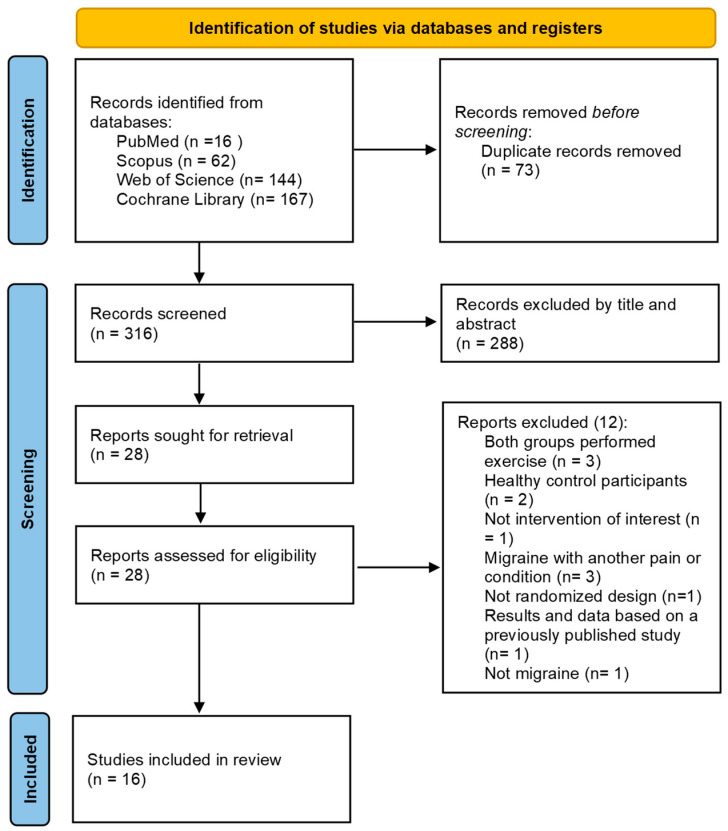
PRISMA 2020 Flowchart [12].

The initial search identified 389 potential articles in PubMed, Scopus, Web of Science, and Cochrane Library. After that, duplicate articles were removed automatically using the Mendeley software (v 1.19.8) (*n* = 73). After the screening by title and abstract, 27 potential reports were reviewed in full text. Supplementary Material S2 shows the 11 excluded reports and their reasons. Finally, 16 articles that met our inclusion criteria were selected [4,23,24,25,26,27,28,29,30,31,32,33,34,35,36,37]. Figure 1 shows the PRISMA 2020 flowchart that describes the whole process of selection for this systematic review.

Table 1 shows the methodological quality assessment of the included studies using the JBI scales for randomized controlled trials. All the included studies have unblinded participants and providers of the interventions. Also, most of the studies did not report whether the allocation of participants was concealed.

### 3.2. Study Design and Population Characteristics

Table 2 shows the main key characteristics of the included and revised studies. All the articles focused on adults with migraines (episodic and chronic) as the target group and examined the impact of exercise on migraines or controlling parameters related to migraine symptoms. Across the 16 included randomized controlled trials [4,23,24,25,26,27,28,29,30,31,32,33,34,35,36,37], a total of 1217 participants were analyzed (considering trials with two arms of interventions, but after the different interventions, a total of 174 participants withdrew from the studies due to various reasons such as dropout, non-compliance, or other external factors. The reason for dropout was only reported in 12 of 16 cases. Only three out of 16 of the included studies reported adverse events during the protocol of this study [24,31,37], but most of the trials did not report this information.

A total of eight studies [23,25,26,27,28,29,30,37] (15 arms of intervention) based their intervention on aerobic exercise, one study [4](two arms) on strength exercise, six studies on yoga [24,32,33,34,35,36], and one on tai-chi [31]. In contrast, most of the participants in the control groups received usual care (*n* = 13), while one study offered dual-task interventions [4], one waitlist [25], one no intervention [31], and one out of 16 education programs [35].

### 3.3. Sensitivity Analysis

One study [24] was identified as an outlier after the sensitivity analysis. Meta-analysis before the sensitivity analysis is displayed in Supplementary Material S3–S6. L’abbe plot shows a homogeneous dispersion of the results, not favoring control or experimental dropout rates. Baujat plot, influence graph, and leave-one-out plot did not present a notable influence of studies with low sample sizes. The funnel plot and Egger’s test (*p* = 0.42) show the absence of publication bias. All these sensitivity analyses are provided in Supplementary Materials S7–S11.

### 3.4. Meta-Analysis of Proportions

**Figure 2:**
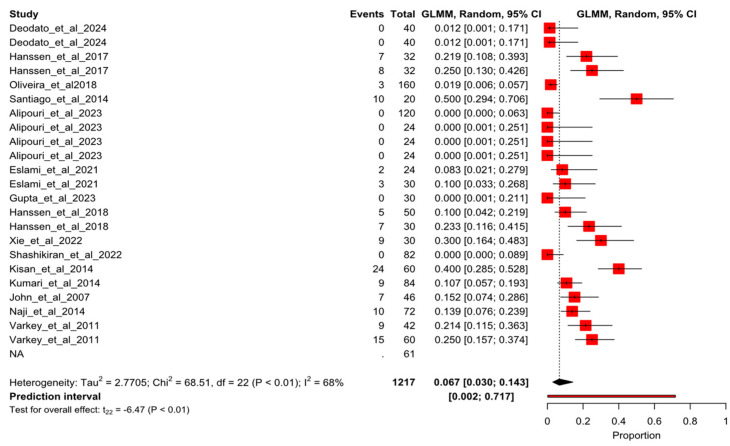
Forest plot of proportion for the overall pooled dropout rate. Deodato et al., 2024 [4], Hanssen et al., 2017 [4], Kumar et al., 2020 [23], Oliveira et al., 2018 [24], Santiago et al., 2014 [25], Alipouri et al., 2023 [26], Eslami et al., 2021 [27], Gupta et al., 2023 [28], Hanssen et al., 2018 [29], Xie et al., 2022 [30], Shashikiran et al., 2022 [31], Kisan et al., 2014 [32], Kumari et al., 2022 [33], John et al., 2007 [34], Naji-Esfahani et al., 2014 [35], Varkey et al., 2011 [36].

**Figure 3:**
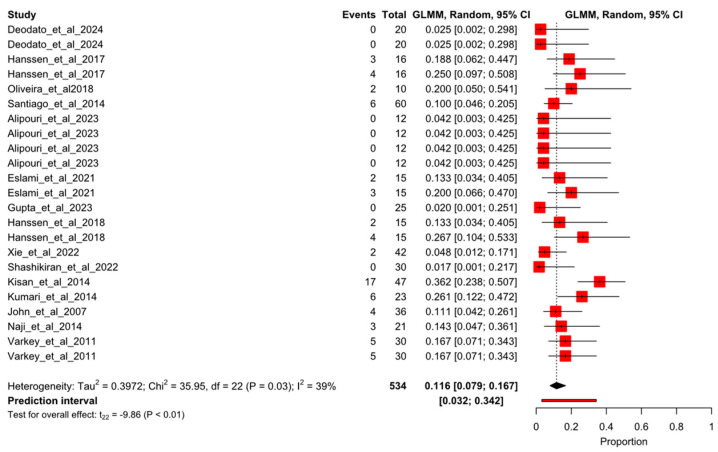
Forest plot of proportion for the experimental pooled dropout rate. Deodato et al., 2024 [4], Hanssen et al., 2017 [4], Kumar et al., 2020 [23], Oliveira et al., 2018 [24], Santiago et al., 2014 [25], Alipouri et al., 2023 [26], Eslami et al., 2021 [27], Gupta et al., 2023 [28], Hanssen et al., 2018 [29], Xie et al., 2022 [30], Shashikiran et al., 2022 [31], Kisan et al., 2014 [32], Kumari et al., 2022 [33], John et al., 2007 [34], Naji-Esfahani et al., 2014 [35], Varkey et al., 2011 [36].

**Figure 4:**
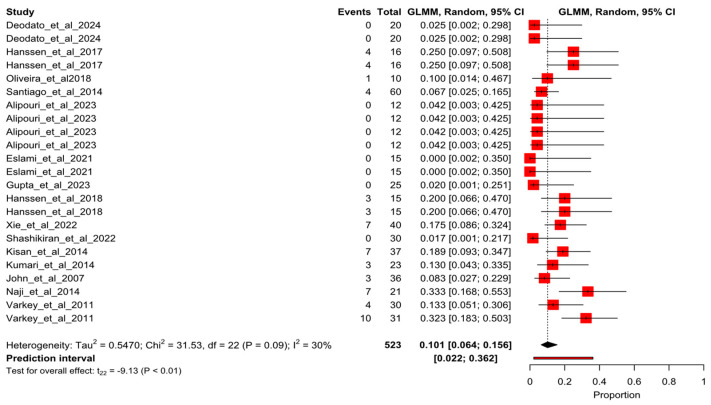
Forest plot of proportion for the control pooled dropout rate. Deodato et al., 2024 [4], Hanssen et al., 2017 [4], Kumar et al., 2020 [23], Oliveira et al., 2018 [24], Santiago et al., 2014 [25], Alipouri et al., 2023 [26], Eslami et al., 2021 [27], Gupta et al., 2023 [28], Hanssen et al., 2018 [29], Xie et al., 2022 [30], Shashikiran et al., 2022 [31], Kisan et al., 2014 [32], Kumari et al., 2022 [33], John et al., 2007 [34], Naji-Esfahani et al., 2014 [35], Varkey et al., 2011 [36].

After the sensitivity analysis, 23 arms of study of 15 randomized controlled trials were meta-analyzed. Figure 2 shows the forest plot for the overall pooled dropout rate of 6.7% (95% CI 3–71.7%; I^2^ = 68%). When dropout events were sorted by study groups, the results were 11.6% (95%CI 7.9–16.7%; I^2^ = 39%) and 10.1% (95%CI 6.4–15.6%; I^2^ = 30%) for exercise groups (Figure 3) and control groups (Figure 4), respectively.

### 3.5. Meta-Analysis of Odds Ratio

**Figure 5:**
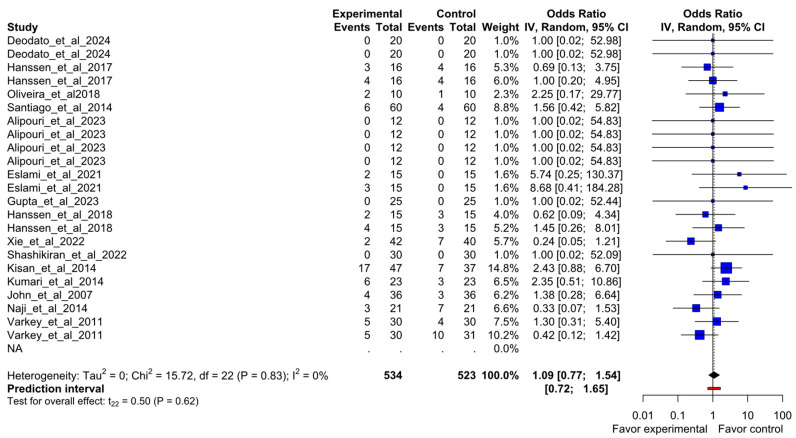
Forest plot of overall odds ratio meta-analysis to compare dropout from study groups of included randomized clinical trials. Deodato et al., 2024 [4], Hanssen et al., 2017 [4], Kumar et al., 2020 [23], Oliveira et al., 2018 [24], Santiago et al., 2014 [25], Alipouri et al., 2023 [26], Eslami et al., 2021 [27], Gupta et al., 2023 [28], Hanssen et al., 2018 [29], Xie et al., 2022 [30], Shashikiran et al., 2022 [31], Kisan et al., 2014 [32], Kumari et al., 2022 [33], John et al., 2007 [34], Naji-Esfahani et al., 2014 [35], Varkey et al., 2011 [36].

After removing the outlier, the overall odds ratio meta-analysis showed no statistical difference in the likelihood of dropout events comparing exercise-based intervention and control comparators with an odds ratio of 1.09 (95%CI 0.77–1.54; I^2^ = 0%). Figure 5 shows the forest plot for this result.

### 3.6. Subgroup Meta-Analysis of Odds Ratio

The forest plots of the subgroup meta-analyses sorted by type of exercise, control intervention, and type of migraine are shown in Supplementary Materials S12–S14.

None of the subgroup analyses showed statistical differences for any types of exercise, control, or type of migraine, except for the migraines with aura. The odds ratio results for migraines with auras showed a lower pooled dropout rate in favor of control participants (participants with migraine aura) OR = 1.18 (95%CI 1.01–1.38; I^2^ = 0%) (Supplementary File S12), but most of the arms of the included studies belonged to Alipouri et al. [27], who did not present any dropout from their participants. This indicates that participants with migraines with aura who received exercise-based interventions had 1.18 times higher odds of dropping out compared to those receiving non-exercise-based comparators.

### 3.7. Certainty of Evidence: GRADE

The GRADE system was conducted for odds ratio meta-analysis. The certainty of evidence was rated as “very low” (Supplementary File S15).

## 4. Discussion

The primary objective of this systematic review with meta-analysis was to assess the current state of attrition in studies comparing exercise-based interventions to other approaches in patients with migraines. This study calculated the overall pooled dropout rate, as well as the dropout rates by intervention group in the included randomized controlled trials. When comparing dropout rates between participants receiving exercise-based interventions and those undergoing other treatments using an odds ratio meta-analysis, the results did not show statistically significant differences, even when subgroup analyses were performed based on exercise type, control group, and migraine type. However, in the case of migraine with aura, a significantly lower pooled dropout rate was observed in favor of control participants. This finding should be interpreted with caution due to the inclusion of multi-arm studies and the small sample size. Additionally, several limitations and substantial heterogeneity were identified across the selected studies, which will be discussed in detail below.

This systematic review highlights the complex relationship between exercise interventions and dropout rates in individuals with migraines. While exercise is widely recognized as a non-pharmacological approach with potential benefits in migraine management, adherence remains a significant challenge [38].

One of the key aspects that may contribute to dropout in exercise-based interventions is the bidirectional behavior of physical activity’s effects on migraine [39]. Exercise presents a complex relationship with migraine. On the one hand, intense physical activity may act as an acute trigger, particularly when performed during the prodromal or attack phases, precipitating migraine episodes [37]. On the other hand, physical inactivity is recognized as a contributing factor that may increase migraine frequency and chronicity [5]. Several studies suggest that moderate, regular exercise may reduce the frequency and severity of migraine attacks by improving vascular function, decreasing inflammation, and modulating pain sensitivity [40,41]. Conversely, there is also evidence that exercise can act as a potential trigger for migraine in susceptible individuals, particularly in high-intensity or prolonged exertion scenarios [42]. This duality may partially explain the inconsistencies in adherence across different interventions, as individuals experiencing symptom exacerbation are more likely to discontinue participation. Regular exercise, when appropriately prescribed and progressively adapted, has been shown to promote peripheral and central desensitization mechanisms, potentially reducing the frequency and severity of migraine attacks over time [40]. This duality highlights the importance of tailoring exercise interventions to each patient’s tolerance and migraine phase.

The type and intensity of exercise also appear to play a role in the attrition or adherence of participants with migraine [43]. Our subgroup analyses indicate that aerobic exercises, particularly those involving high-intensity training, showed slightly higher dropout rates compared to moderate-intensity or low-impact interventions such as yoga and tai chi. These findings align with previous research suggesting that more strenuous exercise regimens may increase the risk of exercise-induced migraine episodes, discouraging continued participation [44]. Additionally, individual variability in response to exercise further complicates adherence patterns, emphasizing the need for personalized exercise prescriptions tailored to migraineurs’ tolerance levels.

Psychosocial factors also influence adherence in migraine populations. Fear of exacerbating migraine symptoms, lack of motivation, and external barriers such as time constraints and scheduling conflicts are common reasons for discontinuation [38]. Studies included in this review reported that participants often cited logistical difficulties, competing obligations, and perceived ineffectiveness as primary reasons for dropout. Addressing these barriers through structured education, gradual exercise progression, and flexible scheduling may enhance adherence and optimize the long-term benefits of exercise for migraine management.

The dropout reasons observed in the included studies highlight important challenges related to participant retention in exercise-based interventions for migraine. Non-adherence to the protocol, personal circumstances, and adverse events were among the most common reasons for study withdrawals. In experimental groups, difficulties attending scheduled sessions, loss of interest, and symptom improvement were frequent factors contributing to dropout. In control groups, dissatisfaction with the assigned intervention, initiation of alternative treatments, and adverse effects related to pharmacological therapies were commonly reported. These findings suggest that retention strategies should be tailored to address the specific challenges faced by each group.

One key issue identified is the variability in how dropout reasons are reported across studies. Some trials provided detailed explanations, while others lacked transparency or grouped different causes under broad categories. This inconsistency limits the ability to draw strong conclusions about the factors influencing participant loss [45,46]. Moreover, while some studies explicitly mentioned adverse events as a cause of withdrawal, others failed to report whether such events occurred. This raises concerns regarding the accuracy of safety assessments in clinical trials involving physical activity interventions. A call for action is necessary to improve the transparency of studies related to dropout in randomized controlled trials focused on exercise and patients with migraine.

Considering that most studies did not document adverse events, we assumed that medical reasons were not directly linked to the interventions. We emphasize the need to standardize the reporting of dropout rates and reasons, as well as adverse events, in accordance with CONSORT guidelines to ensure transparency and improve the reliability of clinical research.

### 4.1. Clinical and Research Implications

The results of this review underscore the need to refine exercise-based interventions for migraine patients. Clinicians should advocate for tailored exercise prescriptions. One possibility for individualized exercise sessions is to start with lower-intensity regimens and gradually increase intensity based on patient tolerance. Additionally, behavioral strategies such as self-monitoring tools may enhance adherence and prevent premature dropout.

Exercise has been proposed to modulate several migraine-related mechanisms, particularly in chronic migraine. Regular physical activity may reduce central sensitization, enhance endorphin release, and improve mood and sleep quality, which are frequently impaired in chronic migraine patients [41]. These adaptations could explain its potential preventive role when appropriately prescribed and sustained over time, but this statement needs to be further studied and assessed to determine how it could influence attrition or retention. Our findings are aligned with recent research supporting the integration of structured exercise, including slow aerobic training and therapeutic physical activity, as part of comprehensive care for migraine. Such interventions may modulate central sensitization and improve quality of life, particularly when combined with pharmacological prophylaxis [47,48,49,50].

From a research perspective, standardizing exercise protocols across studies would improve comparability and facilitate meta-analyses. Moreover, greater consistency in reporting dropout reasons and adverse events is essential to identifying specific factors influencing non-adherence. Long-term follow-up studies are also warranted to assess whether initial dropouts eventually resume physical activity through alternative means.

Future studies should also explore sex, age, and migraine type-based differences in adherence. In addition, future studies should consider the specific migraine phase in which exercise is prescribed or initiated, as physical activity is typically contraindicated during the ictal phase due to heightened sensitivity and discomfort. The presence of comorbidities and simultaneous exposure to other potential migraine triggers should also be taken into account, as these may influence both adherence and dropout independently of the intervention itself. These exploratory studies and analyses will allow clinicians and researchers to elucidate sound conclusions to minimize the possibility of attrition in exercise-based studies, which could be extrapolated to clinical practice. Considering all the points mentioned above, a call for action standardizing the reporting of dropout and adverse events derived from exercise-based intervention in patients with migraines following CONSORT guidelines is required in future research [51]. Also, the use of the CONSORT harms guideline developed in 2022 could be useful for the studies that implemented exercise in patients with migraine since the mechanism of exercise in this condition remains unclear [52].

### 4.2. Limitations

Several limitations should be considered when interpreting the results of this systematic review. First, the included studies exhibited considerable heterogeneity in exercise protocols, migraine subtypes, and control conditions, potentially influencing the generalizability of the findings. Second, dropout reasons were not consistently reported across studies, limiting our ability to conduct a comprehensive analysis of specific factors driving attrition. Third, the reliance on self-reported data for adherence introduces the possibility of recall bias, which may impact the accuracy of dropout classifications. Addressing these limitations in future research will strengthen the evidence base for exercise interventions in migraine populations. Fourth, several studies with more than two arms of interventions were included in the meta-analysis, which is one of the added reasons for considering the results with caution.

## 5. Conclusions

This systematic review and meta-analysis provide a nuanced understanding of dropout rates in exercise-based interventions for migraine. While exercise does not appear to universally lead to higher attrition, individual variability in response, psychosocial barriers, and the nature of the intervention itself play critical roles in adherence. Future studies should focus on optimizing exercise protocols, addressing adherence barriers, and implementing long-term follow-ups to better understand the role of exercise in migraine management. By refining intervention strategies, researchers and clinicians can maximize the therapeutic potential of physical activity while minimizing dropout rates among patients with migraine. Finally, adequate adherence to the CONSORT guidelines in reporting losses and their reasons could help design appropriate retention strategies for studies and interventions based on exercise in patients with migraines.

## Figures and Tables

**Table 1:** Quality assessment using JBI for randomized controlled trials.

Author and Year	I.1	I.2	I.3	I.4	I.5	I.6	I.7	I.8	I.9	I.10	I.11	I.12	I.13
Deodato et al., 2024 [4]	Yes	No	Yes	No	Unclear	Yes	Yes	Yes	Yes	Yes	Yes	Yes	Yes
Hanssen et al., 2017 [4]	Yes	Unclear	Yes	No	No	Yes	Yes	Yes	Yes	Yes	Yes	Yes	Yes
Kumar et al., 2020 [23]	Yes	Yes	Yes	No	No	No	Yes	Yes	Yes	Yes	Yes	Yes	Yes
Oliveira et al., 2018 [24]	Yes	No	Yes	No	No	Yes	No	Yes	Yes	Yes	Yes	Yes	Yes
Santiago et al., 2014 [25]	Yes	Unclear	Yes	No	No	Yes	Yes	Yes	Yes	Yes	Yes	Yes	Yes
Alipouri et al., 2023 [26]	Yes	No	Yes	No	No	Yes	No	Yes	Yes	Yes	Yes	Yes	Yes
Eslami et al., 2021 [27]	Yes	No	Yes	No	No	Yes	Yes	Yes	Yes	Yes	Yes	Yes	Yes
Gupta et al., 2023 [28]	Yes	No	Yes	No	No	Yes	Yes	Yes	Yes	Yes	Yes	Yes	Yes
Hanssen et al., 2018 [29]	Yes	No	Yes	No	No	Yes	No	Yes	Yes	Yes	Yes	Yes	Yes
Xie et al., 2022 [30]	Yes	Yes	Yes	No	No	Yes	Yes	Yes	Yes	Yes	Yes	Yes	Yes
Shashikiran et al., 2022 [31]	Yes	No	Yes	No	No	Yes	Yes	Yes	Yes	Yes	Yes	Yes	Yes
Kisan et al., 2014 [32]	Yes	No	Yes	No	No	Yes	No	Yes	Yes	Yes	Yes	Yes	Yes
Kumari et al., 2022 [33]	Yes	No	Yes	No	No	Yes	Unclear	Yes	Yes	Yes	Yes	Yes	Yes
John et al., 2007 [34]	Yes	No	Yes	No	No	Yes	Unclear	Yes	Yes	Yes	Yes	Yes	Yes
Naji-Esfahani et al., 2014 [35]	Yes	No	Yes	No	No	Yes	Unclear	Yes	Yes	Yes	Yes	Yes	Yes
Varkey et al., 2011 [36]	Yes	No	Yes	No	No	Yes	Unclear	Yes	Yes	Yes	Yes	Yes	Yes
List of items of JBI for RCTs 1. Was true randomization used for the assignment of participants to treatment groups? 2. Was allocation to treatment groups concealed? 3. Were treatment groups similar at baseline? 4. Were participants blind to treatment assignment? 5. Were those delivering the treatment blind to treatment assignment? 6. Were treatment groups treated identically other than the intervention of interest? 7. Were outcome assessors blind to treatment assignment? 8. Were outcomes measured in the same way for treatment groups? 9. Were outcomes measured in a reliable way? 10. Was follow-up complete, and if not, were differences between groups in terms of follow-up adequately described and analyzed? 11. Were participants analyzed in the groups to which they were randomized? 12. Was appropriate statistical analysis used? 13. Was the trial design appropriate, and were any deviations from the standard RCT design accounted for in the conduct and analysis of the trial?													

**Table 2:** Main characteristics and data of interest from included studies.

Author and Year	Sample and Participants. N (Recruited/ Analyzed)/	% Sex (n)/Age	Exercise Intervention	Comparator Intervention	% Overall Retention	% Dropout	Reason of Dropouts	Adverse Events
Deodato et al., 2024 [4] STRENGTH	N = 30 Participants with episodic migraine without aura. EG = 10 CG1 = 10 CG2 = 10	Each group had 2 males and 8 females. Active exercise-only: Mean age 36.5 ± 13.9 years old Cognitive task-only: Mean age 42.7 ± 11.2 years old	3 months, comprising 20 one-hour individual sessions, conducted twice per week + 1 month FLU Lemmens protocol: 20 min warm-up, 30 min strengthening, and 10 min stretching	3 months, comprising 20 one-hour individual sessions, conducted twice per week + 1 month FLU GC1: cognitive task intervention GC2: dual task based on exercise + cognitive task (this group is not considered in the MA)	100% (20/20)	DO:0% (0/20) DEG: 0% (0/10) DCG: 0% (0/10)	N/A	N/A
Hanssen et al., 2017 [4] AEROBICS	N = 48 EG1 = 16 EG2 = 16 CG = 16	Female: 30 (81.1%). Male: 7 (18.9%)|Mean age: 37 years (SD: 10)	12 weeks, with sessions conducted twice weekly EG1: High-Intensity Interval Training (HIT) EG2: Moderate Continuous Training (MCT)	Control group Usual care. Ask to maintain their regular physical activity without structured exercise	77.09% (37/48)	DO:22.92% (11/48) DEG1: 18.75% (3/16) DEG2: 25% (4/16) DCG: 25% (416)	Non-intervention-related injury, pregnancy, non-compliance (reasons not specified by groups)	N/A
Kumar et al., 2020 [23] YOGA	N = 160 EG = 80 CG = 80.	EG: 22 males (27.5%) and 58 females (72.5%). Mean age 30.5 ± 8.01 years. GC: 27 males (33.75%) and 53 females (66.25%) Medical group: Mean age 31.9 ± 8.17 years	Yoga was performed 3 days/week for the first month under supervision, followed by 5 days/week at home for the next 2 months	Medical therapy continued without changes for 3 months	71.25% (114/160)	DO: 28.75% (46/160) DEG: 28.75% (23/80) DCG: 28.75% (23/80)	EG: 6 work issues; 5 studies; 4 personal reasons; 2 headaches improved; 3 moved to another city; 1 mother developed cancer; 1 father ill with CG: 6 worked; 4 phone changes did not come; 3 personal reasons; 2 went to another city to study; 2 FLU from another hospital; 1 headache-free; 1 change to home remedy	EG: 1 adverse event (weight gain) CG: 3 adverse events (2 reported weight gain, 1 reported dryness of mouth).
Oliveira et al., 2018 [24] AEROBICS	N = 20 Women with episodic migraine EG = 10 CG = 10	All participants were women (100%) Mean age: 33.8 ± 10.5 years	12 weeks (3 sessions per week, 30 min per session, plus a 5-min warm-up and cool-down) Aerobic exercise	Waitlist	85% (17/20)	DO: 15% (3/20) DEG: 20% (2/10) DCG: 10% (1/10)	cytokine levels below detection limits in post-intervention analyses	N/A
Santiago et al., 2014 [25] AEROBICS	N = 60 Migraine EG = 30 CG = 30	CG: 88% female, 12% male. Mean age 35 ± 8 years. EG: 79% female, 21% male Mean age 31 ± 9 years	12 weeks of 40-min fast walking outdoors, 3 times per week	Amitriptyline alone: 12 weeks of 25 mg/day medication without exercise	83.3% (50/60)	DO: 16.7% (10/60) DEG: 10% (6/60) DCG: 6.67% (4/60)	CG: 4 participants withdrew due to medication side effects (drowsiness and dry mouth) or non-compliance. EG: 6 participants withdrew due to non-adherence to the exercise program	N/A
Alipouri et al., 2023 [26] AEROBICS	N = 48 EG1 = 12 EG2 = 12 CG1 = 12 CG2 = 12	All participants were men (100%) Mean age: 27.5–29.8 years across groups	Eight weeks, three aerobic exercise sessions per week EG1: Aerobic exercise + Vitamine D EG2: Aerobic exercise + placebo	CG1: Non-exercise groups (Placebo) CG2: Vitamine D Followed their regular physical activity routines for eight weeks.	100%	DO: 0% DEG1: 0% DEG2: 0% DCG1: 0% DCG2: 0%	N/A	N/A
Eslami et al., 2021 [27] AEROBICS	N = 45 Women with migraine EG1 = 15 EG2 = 15 CG = 15	All participants were female (100%) CG: Mean age 32.44 ± 5.74 years EG1: Mean age 38.41 ± 6.20 years EG2: Mean age 25.16 ± 6.08 years	Eight weeks, three sessions per week. Aerobic exercise EG1: Moderate-intensity group of 30–40 min per session at 45–70% VO2max. EG2: High-intensity group of 30–40 min per session at 55–90% VO2max	Usual care The control group was instructed to maintain their regular activities during the intervention.	88.9% (40/45)	DO: 11.1% (5/45) DEG1: 13.33% (2/15) DEG2: 20% (3/15) DCG: 0% (0/15)	EG1: change in work schedule EG2: not interested anymore	N/A
Gupta et al., 2023 [28] AEROBICS	N = 50 EG = 25 CG = 25	EG: 76% female, 24% male. Mean age 26.84 ± 7.61 years. CG: 52% female, 48% male Mean age 31.56 ± 6.86 years	6 weeks, 3 sessions per week, 45 min per session (Aerobic exercise + Therapeutic Pain Neuroscience Education + Conventional treatment).	Usual care for the same 6 weeks	100% (50/50)	DO: 0% (0/50) DEG: 0% (0/25) DCG: 0% (0/25)	N/A	N/A
Hanssen et al., 2018 [29] AEROBIC	N = 45 Episodic migraine EG1 = 15 EG2 = 15 CG = 15	Female: 28 Male: 8 Mean Age: 36 years (±10 years)	12 weeks EG1: High-Intensity Interval Training (HIT) of 4 intervals (4 min at 90–95% HRmax, with 3 min active recovery). EG2: Moderate Continuous Training (MCT) of 45 min at 70% HRmax.	Usual care maintaining usual activities with standard physical activity advice.	80% (36/45)	DO: 20% (9/45) DEG1: 13.33% (2/15) DEG2: 26.67% (4/15) DGC: 20% (3/15)	Non-intervention-related injury, lack of motivation, or personal reasons (Groups not specified)	N/A
Xie et al., 2022 [30] TAICHI	N = 73 EG = 42 CG = 40	All participants were female with episodic migraine EG: Mean 50.9 years (SD ± 10.2). CG: Mean 47.1 years (SD ± 11.8)	12 weeks. 1-h sessions, 5 days per week (3 instructor-led and 2 self-practice sessions) Tai Chi	No structured intervention during this study but maintained usual activities	89.02% (73/82)	DO: 10.98% (9/82) DEG: 4.76% (2/42) DCG: 17.5% (7/40)	EG: time conflicts CG: expectations to be selected for tai chi group	EG: Joint pain (33.8%), Muscle pain (33.3%), Slight sprains (10.3%), Dizziness (5.1%). CG: None
Shashikiran et al., 2022 [31] YOGA	N = 60 EG = 30 CG = 30	Participants included both males and females (specific gender breakdown not provided) Participants aged between 18 and 25 years	Single session of Yoga Nidra (Yogic sleep with conscious awareness)	Single session of supine rest (rest without conscious awareness)	100% (60/60)	DO: 0% (0/60) DEG: 0% (0/30) DCG: 0% (0/30)	N/A	N/A
Kisan et al., 2014 [32] YOGA	N = 84 EG = 37 CG = 47	CG: 11 males, 19 females EG: 9 males, 21 females. CG: Mean 31.27 years (SD ± 8.63) EG: Mean 31.72 years (SD ± 10.77)	(Yoga + Conventional Care): 30 sessions, 6 weeks, 5 days a week. 30 min. Yoga (including loosening exercises, breathing exercises, asanas, and relaxation techniques like Shavasana)	Group CG(Conventional Care): 6 weeks of conventional migraine care, with no structured additional interventions.	71.42% (60/84)	DO: 28.57% (24/84) DEG: 36.17% (17/47) DCG: 18.92% (7/37)	EG: 11 only came for yoga, 6 did not come for post-assessment CG: 3 diary compliance < 7 days, 4 did not come for post-assessment.)	N/A
Kumari et al., 2022 [33] YOGA	N = 43 EG = 23 CG = 20	EG: Female/Male ratio = 11:6. CG: Female/Male ratio = 13:4 EG: Mean 29.88 years (SD ± 6.59) CG: Mean 32.35 years (SD ± 10.19)	12 weeks of Yoga therapy, with 5 sessions per week, each lasting 45–60 min. Combined yoga + Conventional Therapy: Yoga included loosening exercises, pranayama, asanas, and relaxation techniques like Shavasana.	Conventional migraine therapy using NSAIDs, Triptans (acute attacks), and beta-blockers or Topiramate (prophylaxis)	79.1% (34/43)	DO: 20–93% (9/43) DEG: 26.08% (6/23) DCG: 13.04% (3/23)	EG: 3 participants did not complete Yoga therapy; 1 participant conceived during the trial; 2 participants lost to follow-up CG: 3 participants did not attend post-intervention assessments or maintain headache diaries	N/A
John et al., 2007 [34] YOGA	N = 72 EG = 36 CG = 36	EG: Female/Male = 22/10 CG: Female/Male = 27/6 EG: Mean 34.38 years (SD ± 8.74) CG: Mean 34.21 years (SD ± 9.66)	12 weeks of yoga therapy, 5 sessions per week, 60 min per session. Yoga training includes postures, breathing exercises, pranayama, relaxation, and kriya techniques.	Monthly educational sessions on migraine self-care and lifestyle modifications.	90.3% (65/72)	DO: 9.7% (7/72) DEG: 11.11% (4/36) DCG: 8.33% (3/36)	EG: 2 dates unfeasible 1 unsatisfied 1 incomplete data CG: 2 started other therapy 1 moved to city	N/A
Naji-Esfahani et al., 2014 [35] YOGA	N = 42 EG = 21 CG = 21	All participants were female. EG: Mean 35.4 years (SD ± 7.9) CG: Mean 34.9 years (SD ± 8.37)	12 weeks, 3 sessions per week, 75 min per session. Included asanas, pranayama, Shavasana, and Surya Namaskar.	Conventional migraine medication for 12 weeks	76.19% (32/42)	DO: 23.81% (10/42) DEG: 14.29% (3/21) DCG: 33.33% (7/21)	EG: 2 unknown reasons;1 drug side effect CG: 4 worsening their symptoms. 2 refuse to do a blood test. 1-time conflict	N/A
Varkey et al., 2011 [36] AEROBIC	N = 91 EG = 30 CG1 = 31 CG2 = 30	Male: 9 (10%). Female: 82 (90%) Overall: Mean Age: 44.3 years (SD ± 10.6)	12 weeks of indoor cycling sessions, 3 times per week, 40 min per session Aerobic exercise (15 min warm-up, 20 min moderate-to-high intensity, and 5 min cooldown)	CG1: Topiramate Group 12 weeks of topiramate medication, starting at 25 mg/day and gradually increasing to the highest tolerable dose (maximum 200 mg/day) CG2: Relaxation Group 12 weeks of progressive relaxation therapy sessions, once a week, with daily at home practice guided by a CD	79.1%	DO: 20.9% (19/91) DEG: 16.67% (5/30) DCG1: 32.26% (10/31) DCG2: 13.33% (4/30)	EG: Lack of time (*n* = 1); non-compliance (*n* = 4) CG1: Not satisfied with treatment (*n* = 2) Lack of time (*n* = 1) No explanation (*n* = 1) CG2: Refused prophylactic drugs (*n* = 7) Adverse events (*n* = 3)	No adverse events were reported in the Exercise or Relaxation groups. CG: 33% of participants reported adverse events, including paresthesia, fatigue, and mood changes.

DO: dropout overall; DEG: dropout experimental group; DCG: dropout control group; CG: control group; EG: experimental group; N: global sample size, N/A: not applicable; SD: standard deviation.

## Data Availability

Data sharing is not applicable to this article as no datasets were generated or analyzed during the current study.

## Supplementary Materials

The following supporting information can be downloaded at: https://www.mdpi.com/article/10.3390/healthcare13091061/s1, Supplementary Material S1. Search strategy; Supplementary Material S2: list of excluded studies and reasons of exclusion; Suppelementary Material S3. All groups proportion meta-analysis previous to sensitivity analysis; Supplementary Material S4. Propotion meta-analysis of exercise-based interventions previous to sensitivity analysis; Supplementary Material S5. Proportion meta-analysis of comparator previous to sensitivity analysis; Supplementary Material S6. Odds ration meta-analysis previous to sensituvity analysis; Supplementary Material S7. Influence graph; Supplementary Material S8. L'Abbe plot; Supplementary Material S9. Leave-one-out analysis; Supplementary Material S10. Baujat plot; Supplementary Material S11. Funnel plot; Supplementary Material S12. Odds ratio meta-analysis sorted by migraine type; Supplementary Material S13. Odds ratio meta-analysis sorted by type of exercise; Supplementary Material S14. Odds ratio meta-analysis sorted by type of comparator; Supplementary File S15. Certainty of evidence (GRADE) for the odds ratio meta-analysis that compare dropout rates between experimental and control intervention.
