# Supplementary material for: Dropout Rate of Participants in Randomized Controlled Trials Using Different Exercise-Based Interventions in Patients with Migraine. A Systematic Review with Meta-Analysis
Source: Healthcare (Basel). 2025 May 5;13(9):1061. doi: 10.3390/healthcare13091061 (PMC12071463; doi:10.3390/healthcare13091061)
Supplement: Supplementary file 1 [file healthcare-13-01061-s001.zip › 1 REFERENCE AND DESCRIPTION OF SUPPLEMENTARY MATERIALS.pdf]

DESCRIPTION FILE OF SUPPLEMENTARY MATERIALS:

A. *BIBLIOGRAFY INCLUDED IN ALL SUPPLEMENTARY MATERIALS:*

23. Deodato, M.; Granato, A.; Buoite Stella, A.; Martini, M.; Marchetti, E.; Lise, I.; Galmonte, A.; Murena, L.; Manganotti, P. Efficacy of a Dual Task Protocol on Neurophysiological and Clinical Outcomes in Migraine: A Randomized Control Trial. *Neurological Sciences* **2024**, *45*, 4015–4026, doi:10.1007/s10072-024-07611-8.

24. Hanssen, H.; Minghetti, A.; Magon, S.; Rossmeissl, A.; Papadopoulou, A.; Klenk, C.; Schmidt-Trucksäss, A.; Faude, O.; Zahner, L.; Sprenger, T.; et al. Superior Effects of High-Intensity Interval Training vs. Moderate Continuous Training on Arterial Stiffness in Episodic Migraine: A Randomized Controlled Trial. *Front Physiol* **2017**, *8*, doi:10.3389/fphys.2017.01086.

25. Kumar, A.; Bhatia, R.; Sharma, G.; Dhanlika, D.; Vishnubhatla, S.; Singh, R.K.; Dash, D.; Tripathi, M.; Srivastava, M.V.P. Effect of Yoga as Add-on Therapy in Migraine (CONTAIN). *Neurology* **2020**, *94*, doi:10.1212/WNL.0000000000009473.

26. Oliveira, A.B.; Bachi, A.L.L.; Ribeiro, R.T.; Mello, M.T.; Vaisberg, M.; Peres, M.F.P. Exercise-Induced Change in Plasma IL-12p70 Is Linked to Migraine Prevention and Anxiolytic Effects in Treatment-Naïve Women: A Randomized Controlled Trial. *Neuroimmunomodulation* **2017**, *24*, 293–299, doi:10.1159/000487141.

27. Santiago, M.D.S.; Carvalho, D. de S.; Gabbai, A.A.; Pinto, M.M.P.; Moutran, A.R.C.; Villa, T.R. Amitriptyline and Aerobic Exercise or Amitriptyline Alone in the Treatment of Chronic Migraine: A Randomized Comparative Study. *Arq Neuropsiquiatr* **2014**, *72*, 851–855, doi:10.1590/0004-282X20140148.

28. Alipouri, M.; Amiri, E.; Hoseini, R.; Hezarkhani, L.A. Effects of Eight Weeks of Aerobic Exercise and Vitamin D Supplementation on Psychiatric Comorbidities in Men with Migraine and Vitamin D Insufficiency: A Randomized Controlled Clinical Trial. *J Affect Disord* **2023**, *334*, 12–20, doi:10.1016/j.jad.2023.04.108.

29. Eslami, R.; Parnow, A.; Pairo, Z.; Nikolaidis, P.; Knechtle, B. The Effects of Two Different Intensities of Aerobic Training Protocols on Pain and Serum Neuro-Biomarkers in Women Migraineurs: A Randomized Controlled Trail. *Eur J Appl Physiol* **2021**, *121*, 609–620, doi:10.1007/s00421-020-04551-x.

30. Gupta, A.; Kumar, S.; Gupta, A.; Rishi, P. Effect of Aerobic Exercises and Therapeutic Pain Neuroscience Education on Disability, Pain, Head Posture and QOL in Migraine Patients. *Comp Exerc Physiol* **2023**, *19*, 119–126, doi:10.3920/CEP220029.

31. Hanssen, H.; Minghetti, A.; Magon, S.; Rossmeissl, A.; Rasenack, M.; Papadopoulou, A.; Klenk, C.; Faude, O.; Zahner, L.; Sprenger, T.; et al. Effects of Different Endurance Exercise Modalities on Migraine Days and Cerebrovascular Health in Episodic Migraineurs: A Randomized Controlled Trial. *Scand J Med Sci Sports* **2018**, *28*, 1103–1112, doi:10.1111/sms.13023.

32. Xie, Y.J.; Tian, L.; Hui, S.S.-C.; Qin, J.; Gao, Y.; Zhang, D.; Ma, T.; Suen, L.K.P.; Wang, H.H.; Liu, Z.-M.; et al. Efficacy and Feasibility of a 12-Week Tai Chi Training for the Prophylaxis of Episodic Migraine in Hong Kong Chinese Women: A Randomized Controlled Trial. *Front Public Health* **2022**, *10*, doi:10.3389/fpubh.2022.1000594.

33. Shashikiran, H.C.; Shetty, P.; Akshay, R.; Venugopal, A.; Shetty, S. Effect of Yoga Nidra on the Brain Activity in Individuals with Migraine. *Yoga Mimamsa* **2022**, *54*, 18–23, doi:10.4103/ym.ym_35_22.

34. Sathyaprabha, T.; Kisan, R.; Adoor, M.; Nalini, A.; Kutty, B.; ChindandaMurthy, B.; Sujan, M.; Rao, R.; Raju, T. Effect of Yoga on Migraine: A Comprehensive Study Using Clinical Profile and Cardiac Autonomic Functions. *Int J Yoga* **2014**, *7*, 126, doi:10.4103/0973-6131.133891.

35. Kumari, S.; Dhar, M.; Pathania, M.; Kumar, N.; Kulshrestha, P.; Singh, A. Yoga as an Adjuvant Therapy in Management of Migraine- An Open Label Randomised Trial. *J Family Med Prim Care* **2022**, *11*, 5410–5416, doi:10.4103/jfmpc.jfmpc_59_22.

36. John, P.J.; Sharma, N.; Sharma, C.M.; Kankane, A. Effectiveness of Yoga Therapy in the Treatment of Migraine Without Aura: A Randomized Controlled Trial. *Headache: The Journal of Head and Face Pain* **2007**, *47*, 654–661, doi:10.1111/j.1526-4610.2007.00789.x.
37. Hajar Naji-Esfahani, M.Z.S.M.M.V.S.S.H.J. Preventive Effects of a Three-Month Yoga Intervention on Endothelial Function in Patients with Migraine. *Int J Prev Med* **2014**, *5*, 424–429.
38. Varkey, E.; Cider, Å.; Carlsson, J.; Linde, M. Exercise as Migraine Prophylaxis: A Randomized Study Using Relaxation and Topiramate as Controls. *Cephalalgia* **2011**, *31*, 1428–1438, doi:10.1177/0333102411419681.

B. DESCRIPTION OF SUPPLEMENTARY MATERIALS:

1. Supplementary Material 1: The search strategy used in each databases, indicating the results by each database and the date of search.

2. Supplementary Material 2: Listo f excluded studied after the full text read, and the reasons of exclusions.

3. Supplementary Material 3: Proportion meta-analysis of all arms of studies included in the MA, before the sensitivity analysis.

4. Supplementary Material 4: Proportion meta-analysis of experimental interventions data of studies included in the MA, before the sensitivity analysis.

5. Supplementary Material 5: Proportion meta-analysis of control interventions data of studies included in the MA, before the sensitivity analysis.

6. Supplementary Material 6: Odds ratio meta-analysis previous to sensitivity analysis. No studies have been excluded in this case.

7. Supplementary Material 7: Sensitivity analysis represented by a graph, in which the point represents how the studies influence the pooled effects of odds ratio meta-analysis. Kumar et al. was indicated as outlier in red color.

8. Supplementary Material 8: The L'Abbe plot indicates the distribution of studies with respect to the results, showing a tendency towards lower losses when the studies appear in the upper quadrant, towards lower losses in the lower quadrant, and if they are located on the line, there is no greater or lesser drop between groups.

9. Supplementary Material 9: The leave-one-out plot shows how overall heterogeneity would change in the odds ratio meta-analysis as we remove the

studies one by one. It therefore indicates how much heterogeneity each study arm contributes individually to the pooled result or effect size.

10. Supplementary Material 10: Baujat plot shows how the studies influence the overall pooled result and the overall heterogeneity of the meta-analysis
11. Supplementary Material 11: Funnel plot allows you to visually explore whether there are asymmetries in the included studies. The plot helps identify if there is a systematic lack of studies, especially smaller ones with negative or less significant results, which could indicate publication bias. Because no asymmetry was detected in the graph, the presence of publication bias is ruled out.

12. Supplementary Material 12: Subgroup meta-analysis of odds ratios sorted by type of migraine of the different studies. No differences were found by the different subgroups.

13. Supplementary Material 13: Subgroup meta-analysis of odds ratios sorted by type of exercise modalities of the different studies. No differences were found by the different subgroups.

14. Supplementary Material 14: Subgroup meta-analysis of odds ratios sorted by type of control comparators of the different studies. No differences were found by the different subgroups.
15. Supplementary Material 15: GRADE system of odds ratio meta-analysis. The certainty of evidence was rated as "very low" because of the reasons specified in the supplementary table.
