# Supplementary material for: Dropout Rate of Participants in Randomized Controlled Trials Using Different Exercise-Based Interventions in Patients with Migraine. A Systematic Review with Meta-Analysis
Source: Healthcare (Basel). 2025 May 5;13(9):1061. doi: 10.3390/healthcare13091061 (PMC12071463; doi:10.3390/healthcare13091061)
Supplement: Supplementary file 1 [file healthcare-13-01061-s001.zip › Supplementary file 15 GRADE.pdf]

Supplementary File 15. Certainty of evidence (GRADE) for the odds ratio meta-analysis that compare dropout rates between experimental and control intervention.

Summary of findings			Certainty in evidence based on the GRADE approach					
Outcome	Studies (n/k)	Participants (N)	Risk of bias	Inconsistency	Indirectness	Imprecision	Level of evidence	Importance
Odds ratio meta-analysis	15 (24)	1057	Very Serious[1] (-2)	No	Very Serious[2] (-2)	No	Very Low	Critical

Note: GRADE = Grading of Recommendations Assessment, Development and Evaluation.

1. Downgrade two level due to most information is from RCTs with high o some concerns of bias with potential limitations that are likely to lower confidence in the estimate of effect.
2. Downgrade two levels due to large heterogeneity between experimental interventions and measurement of outcomes.
