# Supplementary material for: Dropout Rate of Participants in Randomized Controlled Trials Using Different Exercise-Based Interventions in Patients with Migraine. A Systematic Review with Meta-Analysis
Source: Healthcare (Basel). 2025 May 5;13(9):1061. doi: 10.3390/healthcare13091061 (PMC12071463; doi:10.3390/healthcare13091061)
Supplement: Supplementary file 1 [file healthcare-13-01061-s001.zip › Supplementary Material 1 Search strategy.pdf]

Supplementary Material 1. Search strategy

PubMed (1st March 2025): n=16

(migraine OR headache "chronic migraine" OR "episodic migraine") AND (exercise OR "aerobic exercise" OR yoga OR "mind-body exercise" OR "resistance exercise" OR "strength exercise") AND ("Clinical trial" OR "Randomized controlled trial")

Scopus (1st March 2025): n=62

(migraine OR headache "chronic migraine" OR "episodic migraine") AND (exercise OR "aerobic exercise" OR yoga OR "mind-body exercise" OR "resistance exercise" OR "strength exercise") AND ("Clinical trial" OR "Randomized controlled trial")

Web of Science (1st March 2025): n=144

(migraine OR headache "chronic migraine" OR "episodic migraine") AND (exercise OR "aerobic exercise" OR yoga OR "mind-body exercise" OR "resistance exercise" OR "strength exercise") AND ("Clinical trial" OR "Randomized controlled trial")

Cochrane Library (1st March 2025): n=167

(migraine OR headache "chronic migraine" OR "episodic migraine") AND (exercise OR "aerobic exercise" OR yoga OR "mind-body exercise" OR "resistance exercise" OR "strength exercise") AND ("Clinical trial" OR "Randomized controlled trial")
