# Supplementary material for: Dropout Rate of Participants in Randomized Controlled Trials Using Different Exercise-Based Interventions in Patients with Migraine. A Systematic Review with Meta-Analysis
Source: Healthcare (Basel). 2025 May 5;13(9):1061. doi: 10.3390/healthcare13091061 (PMC12071463; doi:10.3390/healthcare13091061)
Supplement: Supplementary file 1 [file healthcare-13-01061-s001.zip › Supplementary Material 2 List of excluded studies.pdf]

LIST OF EXCLUDED STUDIES AND REASONS OF EXCLUSION

Both groups performed exercise (n=3)

1. Malek EM, Navalta JW, McGinnis GR. Time of Day and Chronotype-Dependent Synchrony Effects Exercise-Induced Reduction in Migraine Load: A Pilot Cross-Over Randomized Trial. Int J Environ Res Public Health. 2023 Jan 23;20(3):2083.
2. Navalta JW, McGinnis GR, Malek EM. Exercise in a natural environment increases program compliance in people with chronic migraine: A pilot cross-over randomized trial. J Bodyw Mov Ther. 2024 Jul;39:116-121.
3. Wanderley D, Valença MM, de Souza Costa Neto JJ, Martins JV, Raposo MCF, de Oliveira DA. Contract-relax technique compared to static stretching in treating migraine in women: A randomized pilot trial. J Bodyw Mov Ther. 2020;24(1):43-49. doi:10.1016/j.jbmt.2019.05.023.

Healthy control participants (n=2)

1. Oliveira AB, Ribeiro RT, Mello MT, Tufik S, Peres MFP. Anandamide Is Related to Clinical and Cardiorespiratory Benefits of Aerobic Exercise Training in Migraine Patients: A Randomized Controlled Clinical Trial. Cannabis Cannabinoid Res. 2019 Dec 9;4(4):275-284.
2. Pairo Z, Parnow A, Sari Aslani P, Mohammadi P, Mirzaeei S, Mohr M. Exercise training reduces systemic inflammation and improves general health status in female migraineurs: a randomised controlled trail. Eur J Appl Physiol. 2024 May;124(5):1397-1408. doi: 10.1007/s00421-023-05371-5.

Not the intervention of interest (n=1)

1. Hisham, S., Manzour, A., Fouad, M.M. et al. Effectiveness of integrated education and relaxation program on migraine-related disability: a randomized controlled trial. Egypt J Neurol Psychiatry Neurosurg 59, 142 (2023).

Migraine with another pain or another conditions (n=3):

1. Krøll L s., Sjödahl Hammarlund C, Gard G, Jensen R h., Bendtsen L. Has aerobic exercise effect on pain perception in persons with migraine and coexisting tension-type headache and neck pain? A randomized, controlled, clinical trial. European Journal of Pain. 2018;22(8):1399-408.
2. Krøll LS, Hammarlund CS, Linde M, Gard G, Jensen RH. The effects of aerobic exercise for persons with migraine and co-existing tension-type headache and neck pain: A randomized, controlled, clinical trial. Cephalalgia. 2018;38(12):1805-16. doi:10.1177/0333102417752119.
3. Kacar HA, Ozkul C, Baran A, Guclu-Gunduz A. Effects of cervical stabilization training in patients with headache: A single-blinded randomized controlled trial. *Eur J Pain*. 2024;28(4):633-648. doi:10.1002/ejp.2208

Not randomized design (n= 1)

1. Luedtke K, Starke W, von Korn K, Szikszay TM, Schwarz A, May A. Neck treatment compared to aerobic exercise in migraine: A preference-based clinical trial. Cephalalgia Reports. 2020;3:1-9. doi:10.1177/2515816320930681

Results and data based on a previously published study (n=1)

1. Zamani Boroujeni M, Marandi SM, Esfarjani F, Sattar M, Shaygannejad V, Javanmard SH. Yoga intervention on blood NO in female migraineurs. Adv Biomed Res. 2015;4:259. doi:10.4103/2277-9175.172995

Not migraine (n= 1):

1. Martín-Vera D, Sánchez-Sierra A, González-de-la-Flor Á, García-Pérez-de-Sevilla G, Domínguez-Balmaseda D, del-Blanco-Muñiz JA. Efficacy of a strength-based exercise program in patients with chronic tension-type headache: A randomized controlled trial. *Front Neurol.* 2023;14:1256303. doi:10.3389/fneur.2023.1256303.
