# Supplementary material for: Dropout Rate of Participants in Randomized Controlled Trials Using Different Exercise-Based Interventions in Patients with Migraine. A Systematic Review with Meta-Analysis
Source: Healthcare (Basel). 2025 May 5;13(9):1061. doi: 10.3390/healthcare13091061 (PMC12071463; doi:10.3390/healthcare13091061)
Supplement: Supplementary file 1 [file healthcare-13-01061-s001.zip › Supplementary material 3_prop_MA__ALL_previous_sensititiv.pdf]

Study	Events	Total	GLMM, Random, 95% CI
Deodato et al 2024a	0	40	0.000 [0.001; 0.167]
Deodato et al 2024b	0	40	0.000 [0.001; 0.167]
Hanssen et al 2017a	7	32	0.219 [0.108; 0.393]
Hanssen et al 2017b	8	32	0.250 [0.130; 0.426]
Kumar et al 2020	46	160	0.287 [0.223; 0.362]
Oliveira et al 2018	3	20	0.150 [0.049; 0.376]
Santiago et al 2014	10	120	0.083 [0.045; 0.148]
Alipouri et al 2023a	0	24	0.000 [0.001; 0.251]
Alipouri et al 2023b	0	24	0.000 [0.001; 0.251]
Alipouri et al 2023c	0	24	0.000 [0.001; 0.251]
Alipouri et al 2023d	0	24	0.000 [0.001; 0.251]
Eslami et al 2021b(hiit)	2	30	0.067 [0.017; 0.231]
Eslami et al 2021a(moderate aerobic)	3	30	0.100 [0.033; 0.268]
Gupta et al 2023	0	50	0.000 [0.001; 0.138]
Hanssen et al 2018a(moderate)	5	30	0.167 [0.071; 0.343]
Hanssen et al 2018a(hiit)	7	30	0.233 [0.116; 0.415]
Xie et al 2022	9	82	0.110 [0.058; 0.198]
Shashikiran et al 2022	0	60	0.000 [0.001; 0.118]
Kisan et al 2014	24	84	0.286 [0.199; 0.391]
Kumari et al 2022	9	46	0.196 [0.105; 0.335]
John et al 2007	7	72	0.097 [0.047; 0.190]
Naji-Esfahani et al 2014	10	42	0.238 [0.133; 0.389]
Varkey et al 2011a	9	60	0.150 [0.080; 0.264]
Varkey et al 2011b	15	61	0.246 [0.154; 0.369]
Heterogeneity: $Tau^2 = 1.6558$; $Chi^2 = 37.23$, df = 23 (P = 0.03); $I^2 = 38\%$		**1217**	**0.079 [0.041; 0.144]**
**Prediction interval**			**[0.005; 0.573]**

Test for overall effect: $t_{23} = -7.47$ ($P < 0.01$)

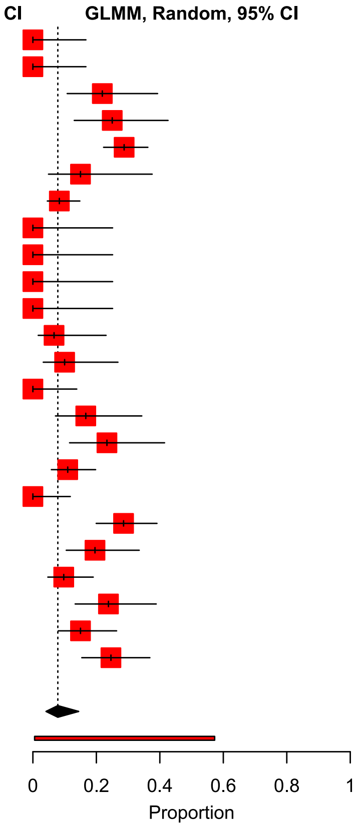

Suppelementary Material 3. All groups proportion meta-analysis previous to sensitivity analysis
