# Supplementary material for: Dropout Rate of Participants in Randomized Controlled Trials Using Different Exercise-Based Interventions in Patients with Migraine. A Systematic Review with Meta-Analysis
Source: Healthcare (Basel). 2025 May 5;13(9):1061. doi: 10.3390/healthcare13091061 (PMC12071463; doi:10.3390/healthcare13091061)
Supplement: Supplementary file 1 [file healthcare-13-01061-s001.zip › Supplementary material 4_prop_MA_EXP_previous_sensit.pdf]

Study	Events	Total	GLMM, Random, 95% CI
Deodato et al 2024a	0	20	0.000 [0.001; 0.287]
Deodato et al 2024b	0	20	0.000 [0.001; 0.287]
Hanssen et al 2017a	3	16	0.188 [0.062; 0.447]
Hanssen et al 2017b	4	16	0.250 [0.097; 0.508]
Kumar et al 2020	23	80	0.287 [0.199; 0.396]
Oliveira et al 2018	2	10	0.200 [0.050; 0.541]
Santiago et al 2014	6	60	0.100 [0.046; 0.205]
Alipouri et al 2023a	0	12	0.000 [0.002; 0.403]
Alipouri et al 2023b	0	12	0.000 [0.002; 0.403]
Alipouri et al 2023c	0	12	0.000 [0.002; 0.403]
Alipouri et al 2023d	0	12	0.000 [0.002; 0.403]
Eslami et al 2021b(hiit)	2	15	0.133 [0.034; 0.405]
Eslami et al 2021a(moderate aerobic)	3	15	0.200 [0.066; 0.470]
Gupta et al 2023	0	25	0.000 [0.001; 0.244]
Hanssen et al 2018a(moderate)	2	15	0.133 [0.034; 0.405]
Hanssen et al 2018a(hiit)	4	15	0.267 [0.104; 0.533]
Xie et al 2022	2	42	0.048 [0.012; 0.171]
Shashikiran et al 2022	0	30	0.000 [0.001; 0.211]
Kisan et al 2014	17	47	0.362 [0.238; 0.507]
Kumari et al 2022	6	23	0.261 [0.122; 0.472]
John et al 2007	4	36	0.111 [0.042; 0.261]
Naji-Esfahani et al 2014	3	21	0.143 [0.047; 0.361]
Varkey et al 2011a	5	30	0.167 [0.071; 0.343]
Varkey et al 2011b	5	30	0.167 [0.071; 0.343]
Heterogeneity: $Tau^2 = 0.8286$; $Chi^2 = 23.63$, df = 23 (P = 0.42); $I^2 = 3\%$		**614**	**0.102 [0.061; 0.165]**
**Prediction interval**			**[0.016; 0.449]**
Test for overall effect: $t_{23} = -8.14$ (P < 0.01)			

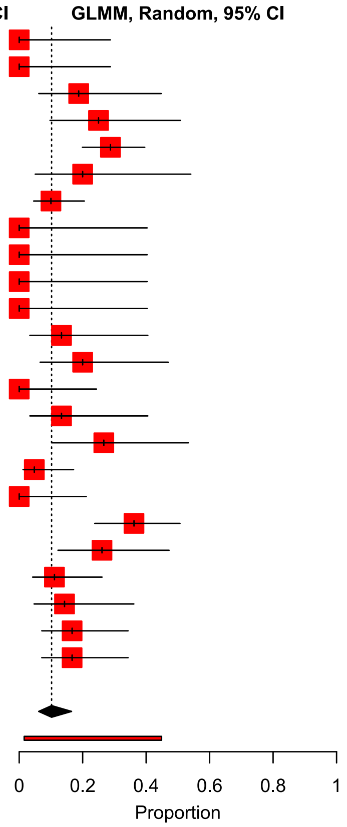

Supplementary Material 4. Propotion meta-analysis of exercise-based interventions previous to sensitivity analysis
