# Supplementary material for: Dropout Rate of Participants in Randomized Controlled Trials Using Different Exercise-Based Interventions in Patients with Migraine. A Systematic Review with Meta-Analysis
Source: Healthcare (Basel). 2025 May 5;13(9):1061. doi: 10.3390/healthcare13091061 (PMC12071463; doi:10.3390/healthcare13091061)
Supplement: Supplementary file 1 [file healthcare-13-01061-s001.zip › Supplementary material 5_prop_MA_CONTR_previous_sensit.pdf]

Study	Events	Total	GLMM, Random, 95% CI
Deodato et al 2024a	0	20	0.000 [0.001; 0.287]
Deodato et al 2024b	0	20	0.000 [0.001; 0.287]
Hanssen et al 2017a	4	16	0.250 [0.097; 0.508]
Hanssen et al 2017b	4	16	0.250 [0.097; 0.508]
Kumar et al 2020	23	80	0.287 [0.199; 0.396]
Oliveira et al 2018	1	10	0.100 [0.014; 0.467]
Santiago et al 2014	4	60	0.067 [0.025; 0.165]
Alipouri et al 2023a	0	12	0.000 [0.002; 0.403]
Alipouri et al 2023b	0	12	0.000 [0.002; 0.403]
Alipouri et al 2023c	0	12	0.000 [0.002; 0.403]
Alipouri et al 2023d	0	12	0.000 [0.002; 0.403]
Eslami et al 2021b(hiit)	0	15	0.000 [0.002; 0.350]
Eslami et al 2021a(moderate aerobic)	0	15	0.000 [0.002; 0.350]
Gupta et al 2023	0	25	0.000 [0.001; 0.244]
Hanssen et al 2018a(moderate)	3	15	0.200 [0.066; 0.470]
Hanssen et al 2018a(hiit)	3	15	0.200 [0.066; 0.470]
Xie et al 2022	7	40	0.175 [0.086; 0.324]
Shashikiran et al 2022	0	30	0.000 [0.001; 0.211]
Kisan et al 2014	7	37	0.189 [0.093; 0.347]
Kumari et al 2022	3	23	0.130 [0.043; 0.335]
John et al 2007	3	36	0.083 [0.027; 0.229]
Naji-Esfahani et al 2014	7	21	0.333 [0.168; 0.553]
Varkey et al 2011a	4	30	0.133 [0.051; 0.306]
Varkey et al 2011b	10	31	0.323 [0.183; 0.503]
Heterogeneity: $Tau^2 = 1.2860$; $Chi^2 = 19.92$, df = 23 (P = 0.65); $I^2 = 0\%$		**603**	**0.082 [0.042; 0.152]**
**Prediction interval**			**[0.008; 0.509]**

Test for overall effect: $t_{23} = -7.12$ ($P < 0.01$)

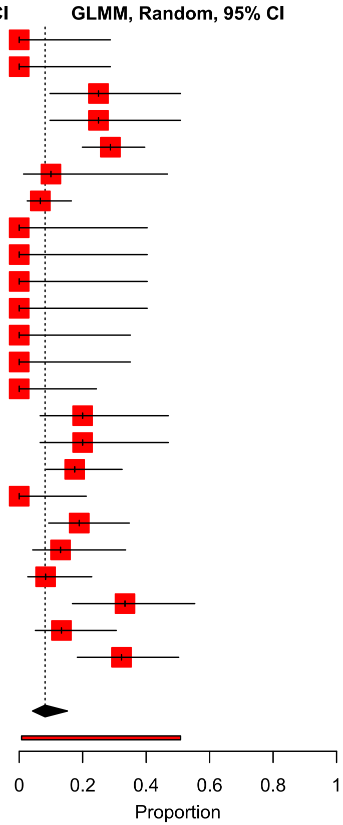

Supplementary Material 5. Proportion meta-analysis of comparator previous to sensitivity analysis
