# Supplementary figures and images for: Dropout Rate of Participants in Randomized Controlled Trials Using Different Exercise-Based Interventions in Patients with Migraine. A Systematic Review with Meta-Analysis
Source: Healthcare (Basel). 2025 May 5;13(9):1061. doi: 10.3390/healthcare13091061 (PMC12071463; doi:10.3390/healthcare13091061)

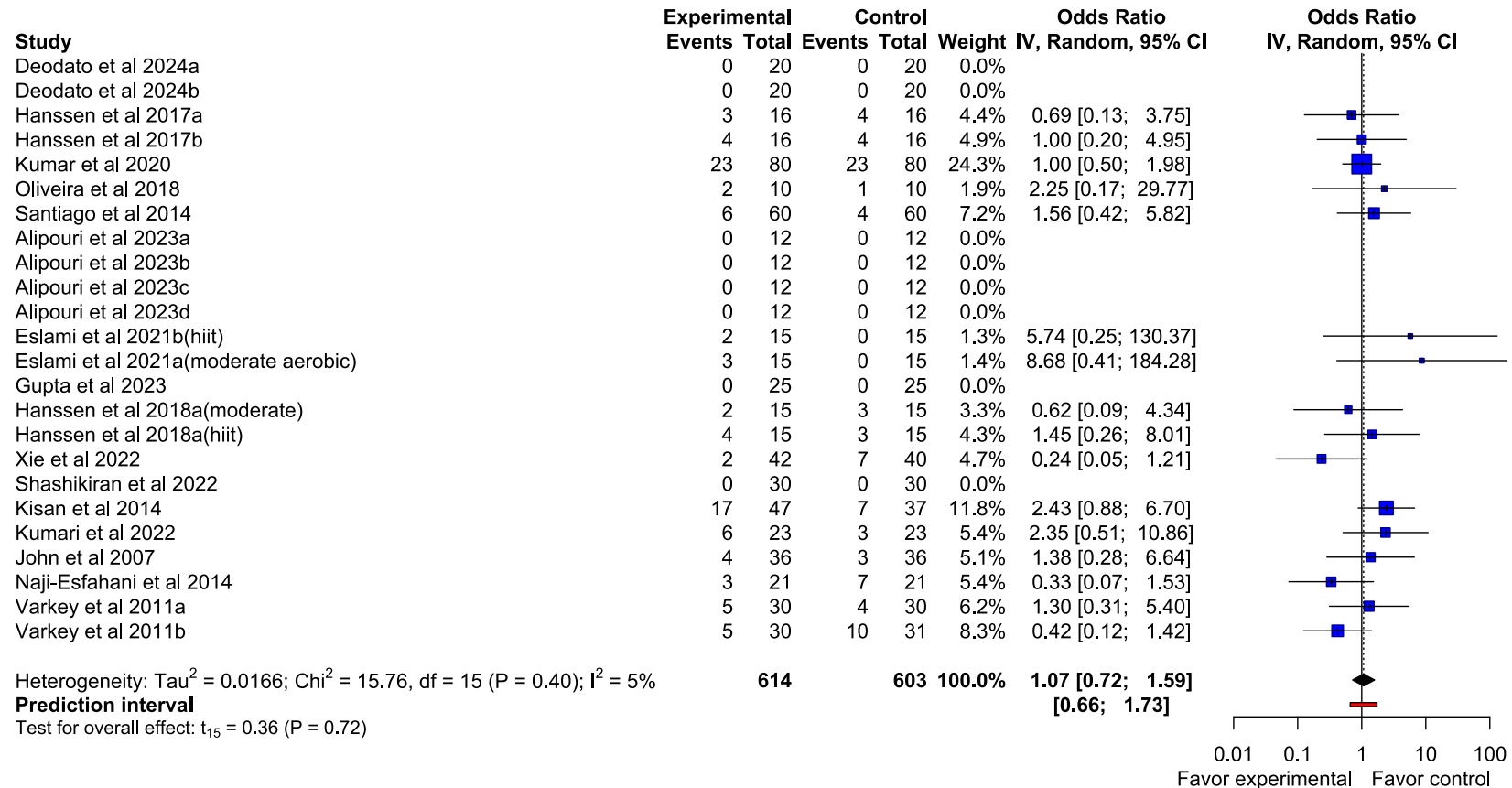

Supplementary Material 6. Odds ration meta-analysis previous to sensitivuty analysis

Supplement: Supplementary file 1 [file healthcare-13-01061-s001.zip › Supplementary material 6_OR_MA_previous_sensiti.pdf]

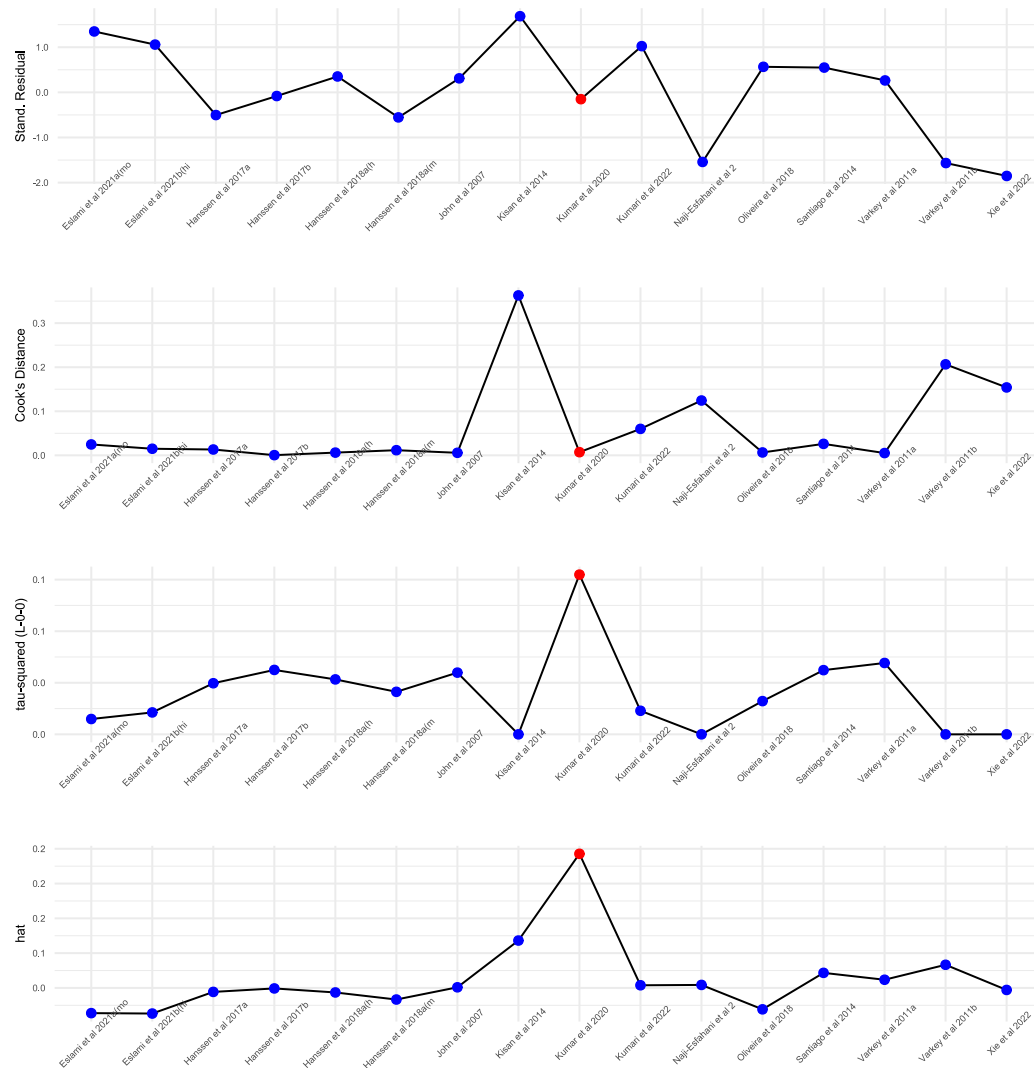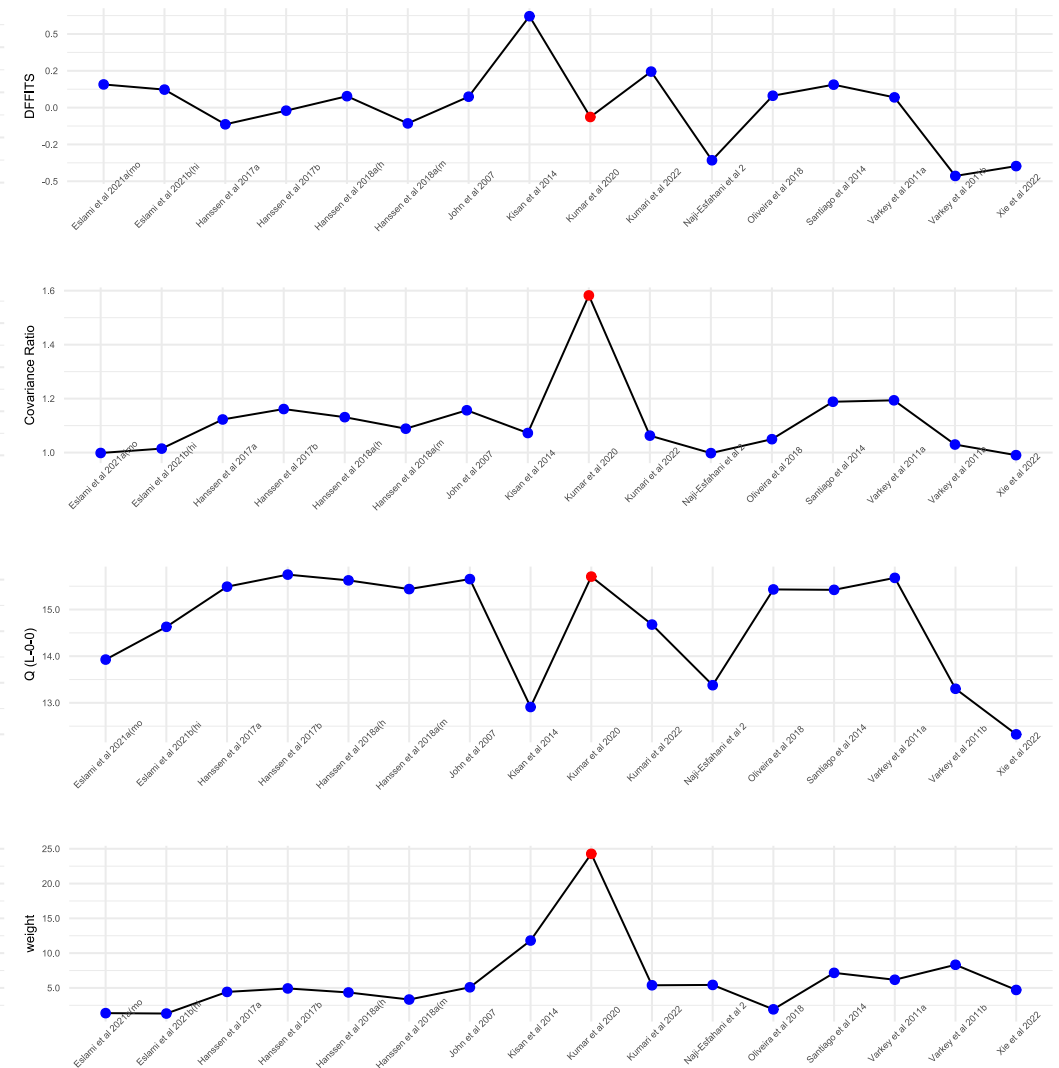

Supplementary Material 7. Influence graph

Supplement: Supplementary file 1 [file healthcare-13-01061-s001.zip › Supplementary Material 7 influence_graph.pdf]

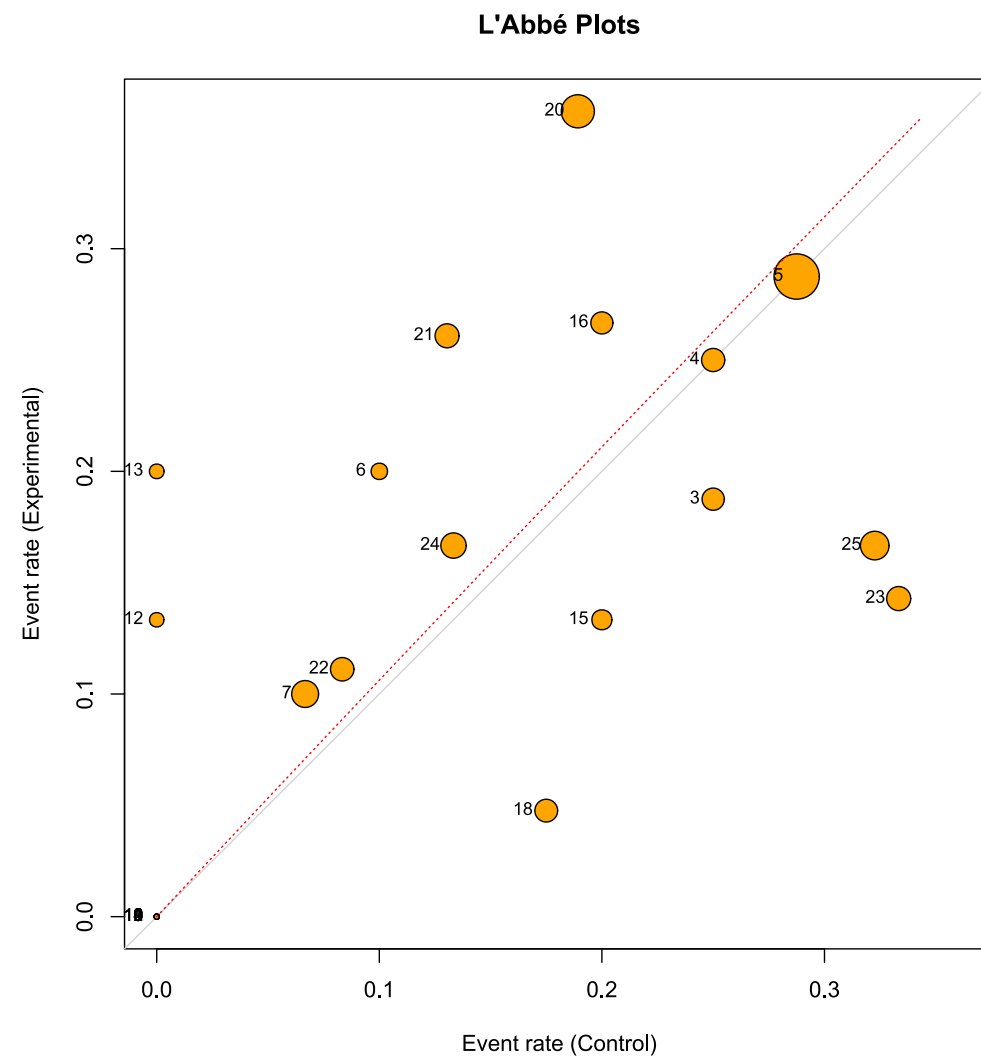

Supplementary Material 8. L'Abbe plot

Supplement: Supplementary file 1 [file healthcare-13-01061-s001.zip › Supplementary Material 8 l abbe.pdf]

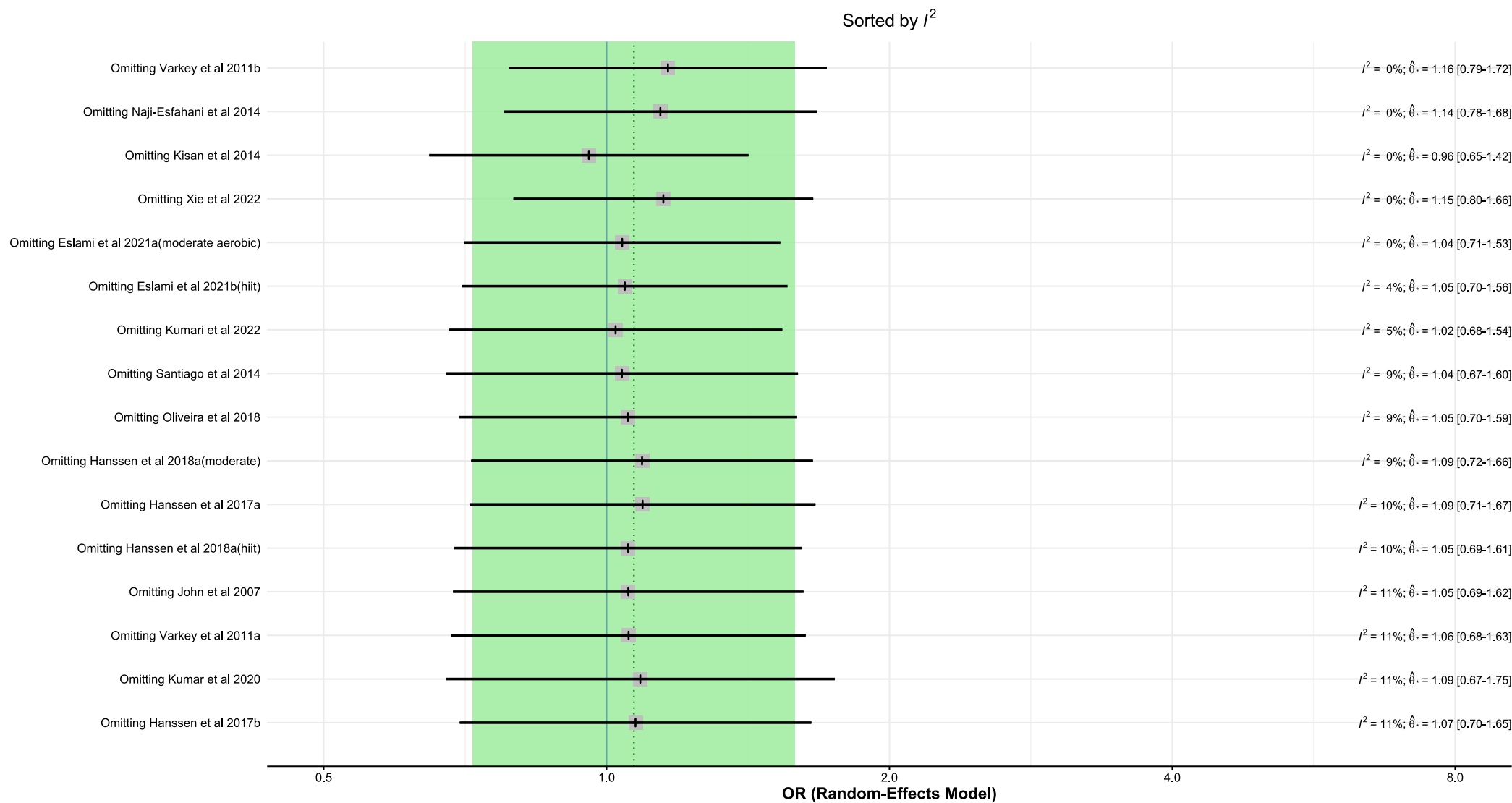

Supplementary Material 9. Leave-one-out analysis

Supplement: Supplementary file 1 [file healthcare-13-01061-s001.zip › Supplementary Material 9 leave_one_out.pdf]

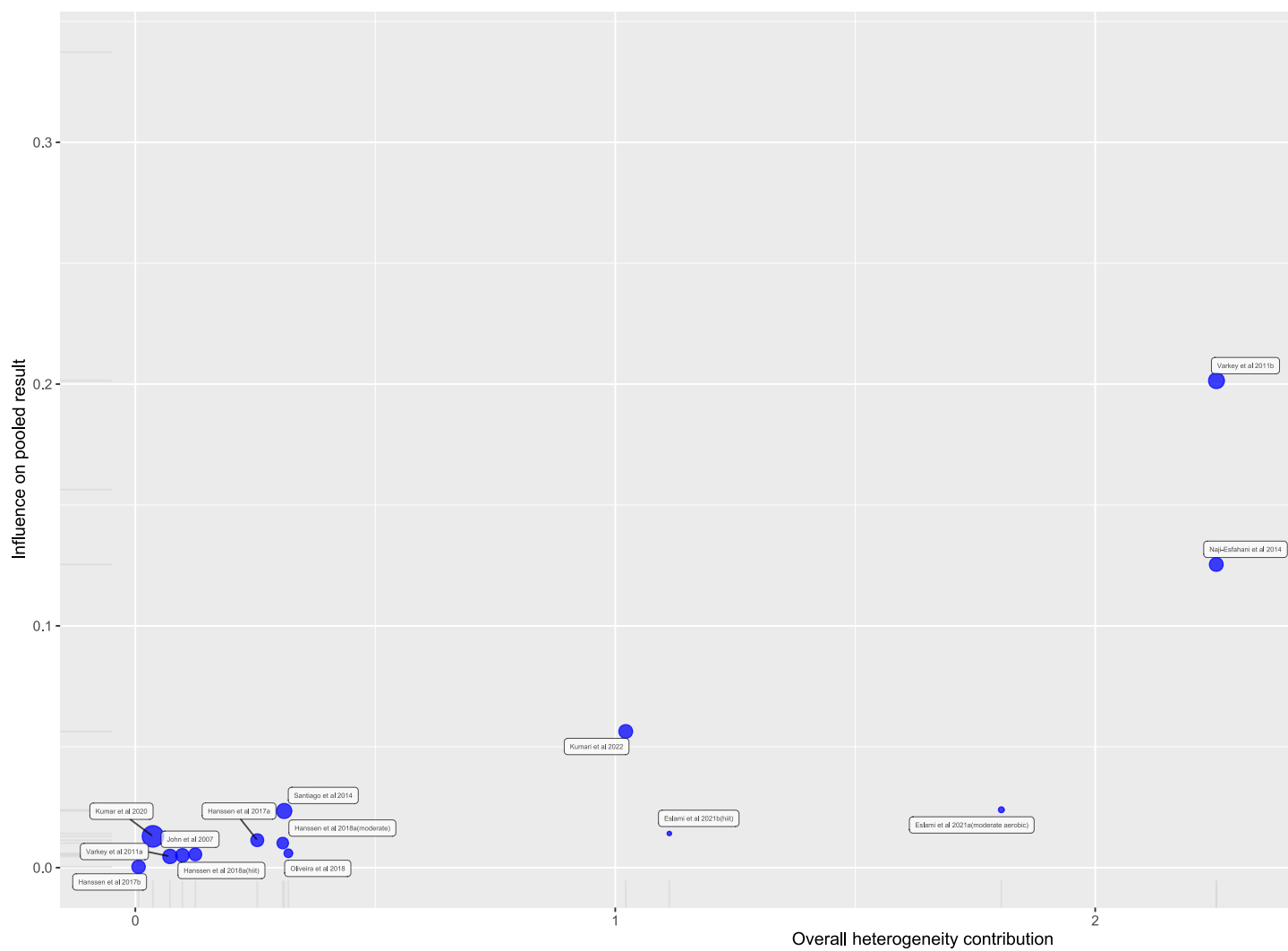

Supplementary Material 10. Baujat plot

Supplement: Supplementary file 1 [file healthcare-13-01061-s001.zip › Supplementary Material 10 Baujat.pdf]

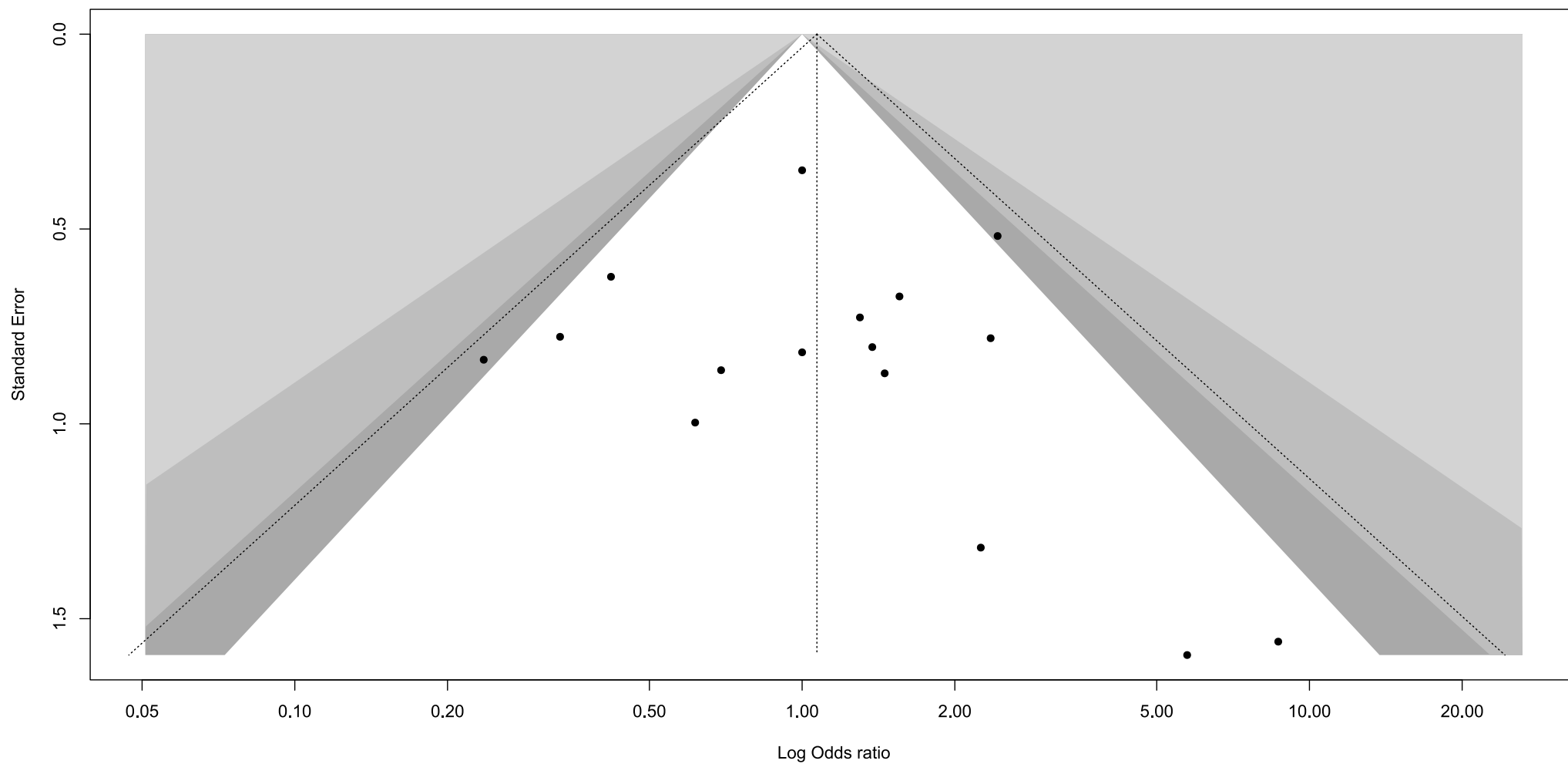

Supplementary Material 11. Funnel plot

Supplement: Supplementary file 1 [file healthcare-13-01061-s001.zip › Supplementary Material 11 Funnel plot.pdf]

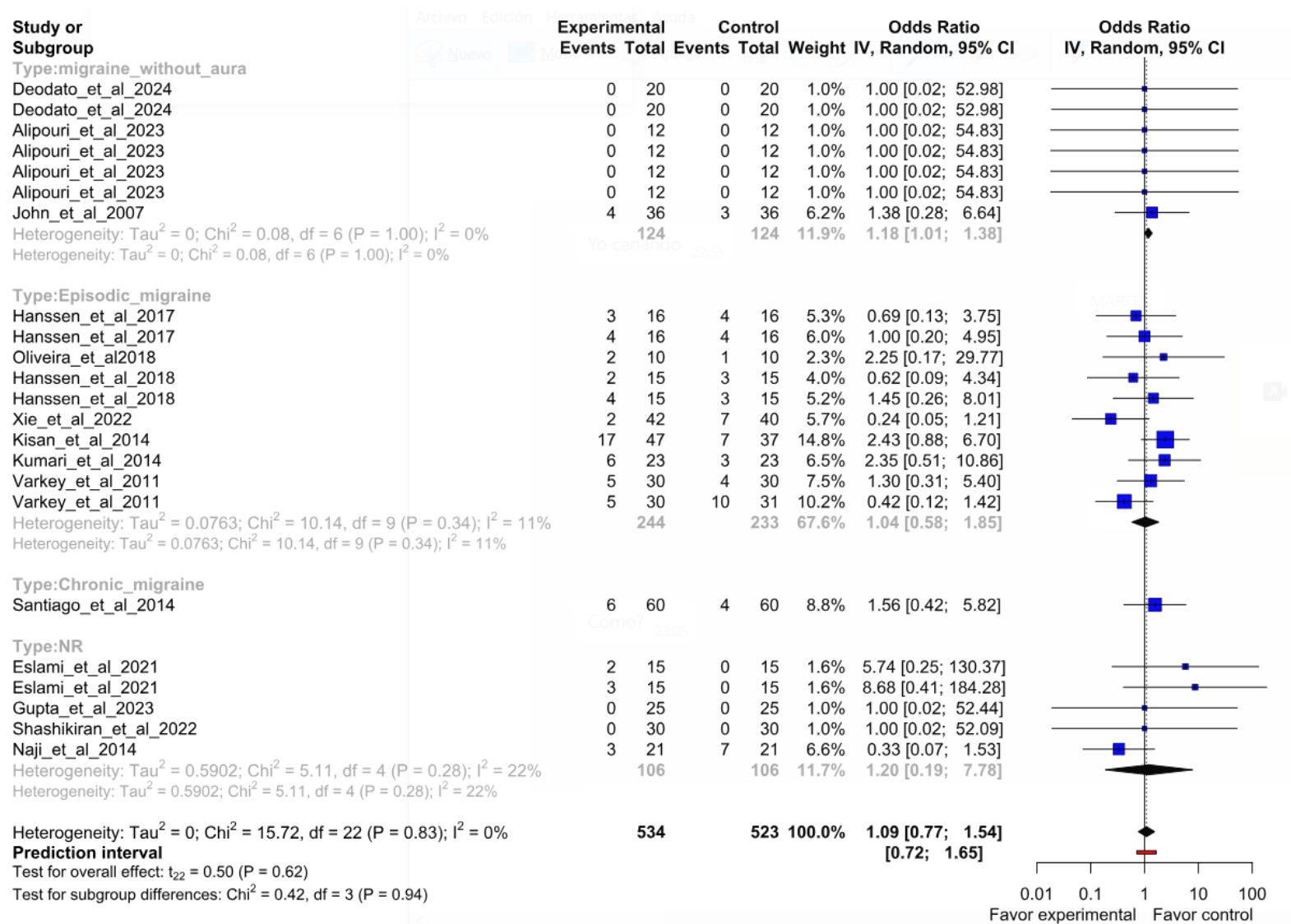

Supplementary Material 12. Odds ratio meta-analysis sorted by migraine type

Supplement: Supplementary file 1 [file healthcare-13-01061-s001.zip › Supplementary Material 12 MA_subgroup_migraine.pdf]

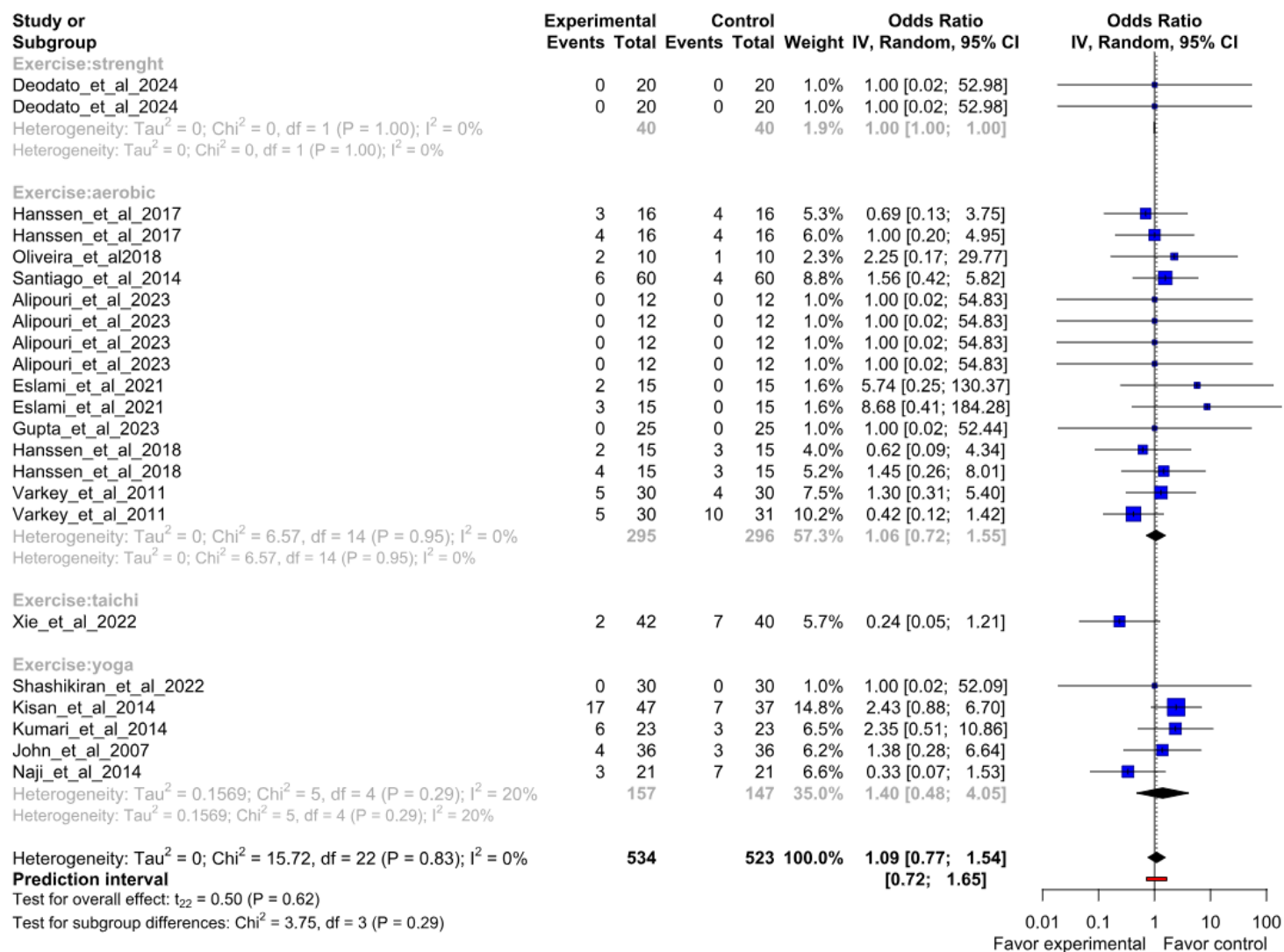

Supplementary Material 13. Odds ratio meta-analysis sorted by type of exercise

Supplement: Supplementary file 1 [file healthcare-13-01061-s001.zip › Supplementary Material 13 MA_subgroup_exercise.pdf]

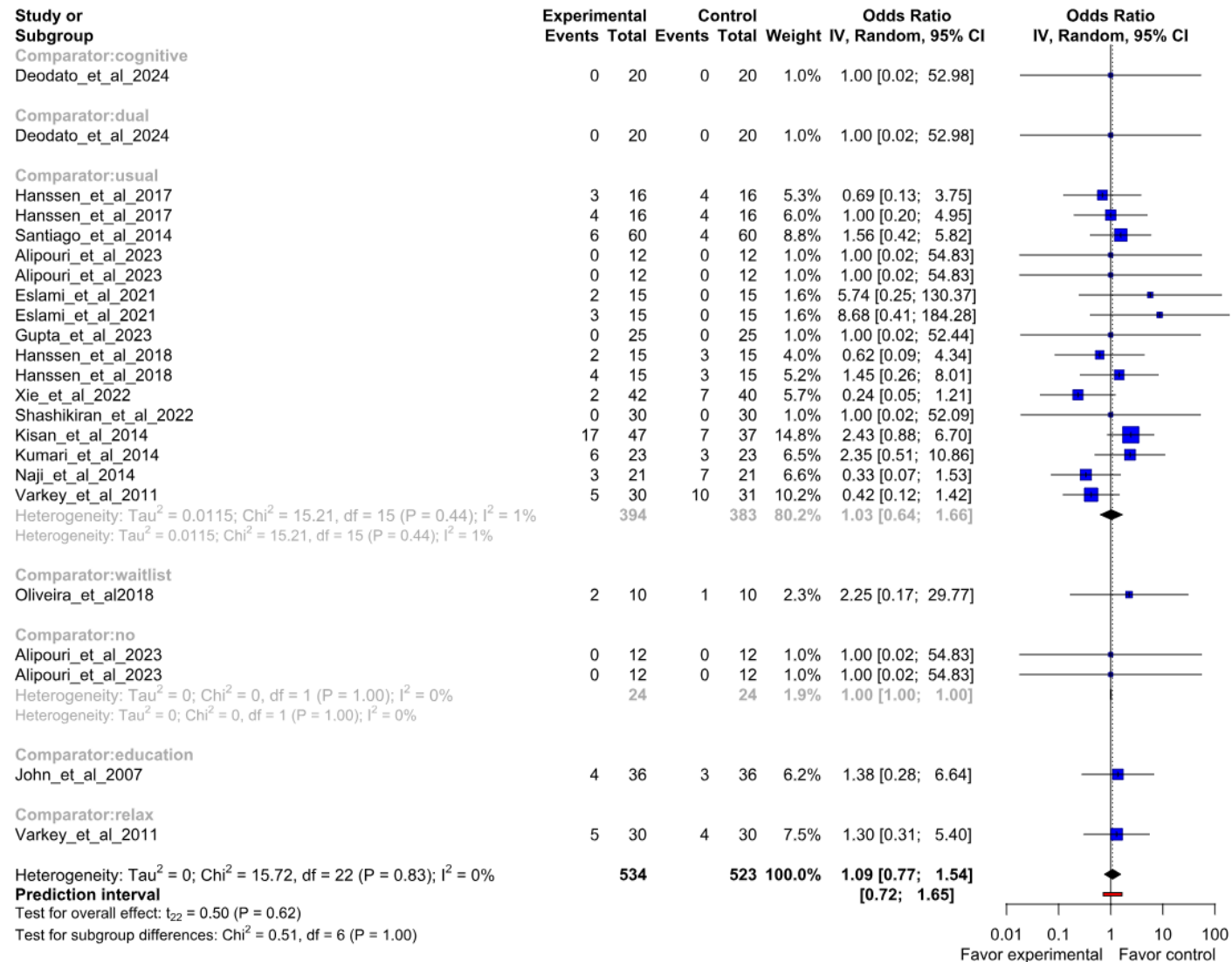

Supplementary Material 14. Odds ratio meta-analysis sorted by type of comparator

Supplement: Supplementary file 1 [file healthcare-13-01061-s001.zip › Supplementary Material 14 MA_subgroup_control.pdf]
